# Regulatory dissection of the *CBX5* and *hnRNPA1* bi-directional promoter in human breast cancer cells reveals novel transcript variants differentially associated with HP1α down-regulation in metastatic cells

**DOI:** 10.1186/s12885-016-2059-x

**Published:** 2016-01-20

**Authors:** Johan Vad-Nielsen, Kristine Raaby Jakobsen, Tina Fuglsang Daugaard, Rune Thomsen, Anja Brügmann, Boe Sandahl Sørensen, Anders Lade Nielsen

**Affiliations:** Department of Biomedicine, The Bartholin building, Aarhus University, DK-8000 Aarhus C, Denmark; Department of Clinical-Biochemistry, Aarhus University Hospital, Aarhus, Denmark; Department of Pathology, Aalborg University Hospital, Aalborg, Denmark

**Keywords:** Metastasis, Heterochromatin, Cell invasion, Epigenetics, Transcriptional regulation

## Abstract

**Background:**

The three members of the human heterochromatin protein 1 (HP1) family of proteins, HP1α, HP1β, and HPγ, are involved in chromatin packing and epigenetic gene regulation. HP1α is encoded from the *CBX5* gene and is a suppressor of metastasis. *CBX5* is down-regulated at the transcriptional and protein level in metastatic compared to non-metastatic breast cancer. *CBX5* shares a bi-directional promoter structure with the *hnRNPA1* gene. But whereas *CBX5* expression is down-regulated in metastatic cells, *hnRNAP1* expression is constant. Here, we address the regulation of *CBX5* in human breast cancer.

**Methods:**

Transient transfection and transposon mediated integration of dual-reporter mini-genes containing the bi-directional *hnRNPA1* and *CBX5* promoter was performed to investigate transcriptional regulation in breast cancer cell lines. Bioinformatics and functional analysis were performed to characterize transcriptional events specifically regulating *CBX5* expression. TSA treatment and Chromatin Immunoprecipitation (ChIP) were performed to investigate the chromatin structure along *CBX5* in breast cancer cells. Finally, expression of *hnRNPA1* and *CBX5* mRNA isoforms were measured by quantitative reverse transcriptase PCR (qRT-PCR) in breast cancer tissue samples.

**Results:**

We demonstrate that an *hnRNPA1* and *CBX5* bi-directional core promoter fragment does not comprise intrinsic capacity for specific *CBX5* down-regulation in metastatic cells. Characterization of transcriptional events in the 20 kb *CBX5* intron 1 revealed existence of several novel *CBX5* transcripts. Two of these encode consensus HP1α protein but used autonomous promoters in intron 1 by which HP1α expression could be de-coupled from the bi-directional promoter. In addition, another *CBX5* transcriptional isoform, *STET*, was discovered. This transcript includes *CBX5* exon 1 and part of intron 1 sequences but lacks inclusion of HP1α encoding exons. Inverse correlation between *STET* and HP1α coding *CBX5* mRNA expression was observed in breast cancer cell lines and tissue samples from breast cancer patients.

**Conclusion:**

We find that HP1α is down-regulated in a mechanism involving *CBX5* promoter downstream sequences and that regulation through alternative polyadenylation and splicing generates a transcript, *STET,* with potential importance in carcinogenesis.

**Electronic supplementary material:**

The online version of this article (doi:10.1186/s12885-016-2059-x) contains supplementary material, which is available to authorized users.

## Background

The heterochromatin protein 1 (HP1) family was first identified in *Drosophila melanogaster* as essential components of pericentric heterochromatin and shown to be implicated in chromatin compaction and epigenetic repression of gene expression [1]. In mammalian cells, the HP1 family is composed of three distinct genes: *CBX5*, *CBX1*, and *CBX3* encoding the highly conserved proteins: HP1α, HP1β, and HP1γ [2–5]. The HP1 proteins consist of an N-terminal chromo domain (CD) and a structurally similar C-terminal chromo shadow domain (CSD) separated by a flexible hinge domain [6, 7]. The HP1 proteins have distinct chromatin distributions with HP1α present mainly in heterochromatin, HP1β in both hetero- and euchromatin, and HP1γ primarily located in euchromatin [5, 8, 9]. Tethering HP1 proteins to chromatin through the CD, CSD or heterologous DNA-binding domains results in transcriptional repression *in cis* [8, 10]. The CD mediates HP1 binding to chromatin through specific interactions with di- and tri-methylated lysine 9 on the H3 histone tail (H3K9me2/3). Furthermore, the affinity for CD binding increases proportionally with the degree of methylation [8, 11, 12]. The CD also interacts with the tail of linker histone H1.4 methylated on lysine 26 which participates in further chromatin compaction [13]. The CSD functions as a HP1 protein-protein dimerization domain forming homo- and hetero-dimers [8, 14, 15]. The CSD dimeric structure is also an interaction platform for additional proteins through the core amino acid sequence PXVXL (X = any amino acid) [14, 15]. Many different types of proteins containing PXVXL motifs have been shown to interact with HP1 proteins through the CSD [4, 5, 16–20]. However, there are proteins that associate with the CSD of HP1 through alternative sequence motifs [10, 21, 22]. Notably, the CSD also interacts with the first helix of the histone fold of H3 to a PXVXL-like motif and this H3 region is involved in chromatin remodeling [23–26]. The hinge region of HP1 contributes to chromatin association through interactions with histone H1 and RNA. Through this interaction, RNA components are thought to be important in the maintenance and localization of HP1 proteins along specific sites at the genome, e.g. for HP1α pericentric heterochromatin localization [8, 27–30]. When HP1 is bound to di- or tri-methylated H3K9 through the CD, subsequent recruitment of SUV39h1 causes adjacent H3K9 residues to become methylated. This creates new binding sites for additional HP1 proteins, which, in turn, will further recruit SUV39h1 proteins. This mechanism explains how HP1 modulates the spread of heterochromatin into neighboring euchromatin, a phenomenon known as position effect variegation (PEV) [31–33]. PEV is suppressed with decreased HP1 expression and enhanced with increased HP1 expression [32, 33].

In breast cancer, the expression level of *CBX5* and encoded HP1α correlates with both clinical outcome in terms of patient survival and clinical data in terms of tumor size and stage of this disease [34]. Tumor cells from primary breast carcinomas exhibit higher expression levels of HP1α encoding mRNA and protein compared to normal breast tissue [34]. Moreover, HP1α encoding mRNA and protein have also been shown to be down-regulated in highly invasive breast cancer cell lines (e.g. HS578T and MDA-MB-231) compared to poorly invasive breast cancer cell lines (e.g. T47D and MCF7) while HP1β and HP1γ were relative equally expressed [20, 35–37]. Immunohistochemical analysis of *in vivo* breast cancer samples showed that HP1α expression was reduced in metastatic cells relative to the primary tumor corroborating the cell line findings [36]. Following RNAi-mediated knockdown of HP1α, poorly invasive MCF7 cells have increased invasive potential. Conversely, highly invasive MDA-MB-231 cells loose invasive potential following ectopic HP1α expression [36, 38]. Based on these data, HP1α is defined as a metastasis suppressor, which in contrast to tumor suppressors is defined as factors being able to suppress metastasis without affecting the growth of the tumor [20, 36, 38, 39].

Analysis of the transcriptional regulation of *CBX5* in breast cancer cells have been performed with a resulting mapping of *cis*-elements and *trans*-factors [40, 41]. *CBX5* is orientated in a “head-to-head” bi-directional arrangement with *hnRNPA1*. The *hnRNPA1* encoded protein belongs to the A/B subfamily of heterogeneous nuclear ribonucleoproteins involved in the packaging of pre-mRNA into hnRNP particles, transport of poly adenylated mRNA from the nucleus to the cytoplasm, and may modulate splice site selection [42]. *CBX5* and *hnRNPA1* shares a 0.6 kb promoter sequence including binding sites for E2F and MYC-family transcription factors. Introduction of mutation in a USF/C-MYC recognition site upstream for the *CBX5* transcriptional start site diminished differential expression in invasive versus poorly invasive breast cancer cells [40]. Also, *CBX5* promoter binding of the transcription factor YY1 is involved in regulating the differential expression levels in breast cancer cells [41]. The decrease in *CBX5* expression level in metastatic breast cancer cells correlates with decreased presence of H3K36me3, RNA polymerase II (Pol-II), and basal transcription factors at the promoter [37].

In this study, we find the differential expression of *CBX5* in metastatic versus non-metastatic breast cancer cells requires a decoupling from the bi-directional promoter architecture of *CBX5* and *hnRNPA1*, and investigate sequences downstream of the *CBX5* promoter as possible mediators hereof.

## Methods

### Cell lines

MCF-7 (non-invasive breast cancer cells), MDA-MB-231 (highly invasive breast cancer cells), HEMC (Primary human mammary epithelial cells) and HeLa (cervical cancer cells) were grown in Dulbecco’s Modified Eagle’s Medium DMEM (Lonza) supplemented with 10 % fetal bovine serum, 1 % penicillin and 1 % glutamine. The cells were kept in a CO_2_-incubator with 5 % CO_2_ at 37 °C. The MCF7 and MDA-MB-231 cell lines were purchased from American Type Culture Collection, USA and HEMC from Life Technologies. For TSA treatment of cells 3x10^5^ MCF7 and MDA-MB-231 cells were seeded in 6 - well plates the day before treatment. At the day of treatment, the media was replaced with growth media containing 1 μM TSA (Sigma) from a stock of 1 mM dissolved in a DMSO solution of 1:3.3. As a control, separate cells where given growth media containing the same amount of DMSO. The cells were harvested after 24 hours. mRNA stability in the MDA-MB-231 and MCF7 cells lines was examined by treating cells with Actinomycin D (Sigma), which inhibits *de novo* Pol-II transcription. 24 hours prior to treatment, 5x10^5^ cells were seeded in 25 cm^2^ flasks to reach a confluence of 80 % at the time of treatment. Cells were added fresh DMEM growth media with Actinomycin D diluted in DMSO (1:3) to a final concentration of 10 μg/ml. Cells from one 25 cm^2^ flask were harvested after 0, 2, 4, 8, 12 and 24 hours, by washing twice with PBS and scraping in 1 ml Tri Reagent™ (Sigma) and subjected to RNA purification.

### Breast cancer tissue

Breast tissue specimens were obtained from primary breast cancer surgical procedures as described [43]. The Regional Ethics Committee Northern Jutland, Denmark approved the study (N-20070047), and signed informed consent was obtained from each patient.

### RNA and cDNA

RNA purification was performed using Tri Reagent™ (Sigma). The suspension was transferred to RNAse-free eppendorf tubes and incubated for 5 minutes. 200 μl chloroform (Merck) was added per ml Tri Reagent and incubated for 10 minutes. After centrifugation at 12,000xg for 15 minutes at 4 °C, the upper RNA-containing phase was transferred to RNAse-free eppendorf tubes. 500 μl isopropanol (Merck) and 2 μl glycogen (Sigma) was added followed by centrifugation at 12,000xg for 30 minutes at 4 °C. The pellet was washed in 75 % RNAse-free ethanol and dissolved in 50 μl DEPC H_2_O and stored at −20 °C. RNA concentration was measured using a Thermo Scientific Nanodrop™ spectrophotometer. RNA integrity was confirmed by running samples on 1 % agarose gels with added ethidium bromide (AppliChem). For cell lines cDNA was synthesized from 0.5 μg RNA using the BIO-RAD iScript™ cDNA Synthesis kit containing a mix of oligo(dT) and random hexamer primers was used. After synthesis the cDNA product was diluted with redistilled water to a total volume of 100 μl and stored at −20 °C. For breast cancer samples, cDNA was synthesized from RNA previously isolated from primary normal breast tissue, breast carcinomas and lymph node metastases [43, 44]. cDNA was synthesized in a 20 μl reaction mix including 50 μmol/L Oligo(dT), reverse transcriptase (50 units/μL), RNase inhibitors (20 units/μL), 0.4 mmol/L of each dNTP, 1xPCR buffer, and 25 mmol/L MgCL2 (all from Applied Biosystems Inc., CA, USA). Reverse transcription was performed on the Perkin-Elmer GeneAmp PCR System 9600 Thermal Cycler (PerkinElmer Inc., MA, USA) with the profile: 42 **°**C for 30 minutes, 99 **°**C for 5 minutes and 4 **°**C until samples had cooled. cDNA was stored at **−**20 **°**C until further use.

For rapid amplification of cDNA 3′-ends (3′RACE) the first synthesis reaction utilized an oligo(dT)V primer with anchor sequence (GCGGAATTCGGATCCCTCGAGTTTTTTTTTTTTTTTTTTTV*, *V denotes G, C or A). cDNA was synthesized using 2 μg total RNA, 1 μl oligo(dT)V primer (50 pmol), 1 μl dNTP mix 10 mM (Qiagen), and nuclease-free water to a final volume of 13 μl. After incubation at 65 °C for 5 minutes, 4 μl First Strand Buffer (Invitrogen) and 2 μl DTT (Invitrogen) was added. Following incubation at 42 °C for 2 minutes, samples were added 1 μl (15U) Superscript II Reverse Transcriptase (Invitrogen) to a total volume of 20 μl and further incubated at 42 °C for 50 minutes. The PCR reaction was conducted with 5 μl of synthesized cDNA template, 10 pmol of target cDNA forward primer (*CBX5* exon1 forward, GCAGACGTTAGCGTGAGTG) and 10 pmol of reverse oligo(dT)-r primer (GCGGAATTCGGATCCCTCGAGTT). A nested PCR was performed using reverse oligo(dT)-r primer and a target cDNA forward primer located downstream of the forward primer (*STET* nested forward, TGTAAGCCACTCGAAGCCACA). PCR products of interest were extracted after gel electrophoresis and sequenced.

### Quantitative reverse transcriptase polymerase chain reaction (RT-qPCR)

For cell lines, RT-qPCR was performed in a total reaction volume of 10 μl including 1 μl cDNA, 5 μl Roche LightCycler® 480 SYBR Green I Master enzyme (Roche), 10 pmol of both forward and reverse primer and double distilled water up to 10 μl. A LightCycler® 480 (Roche) was used with a PCR profile of 10 sec denaturation at 95 °C, 20 sec annealing at 95 °C and 1 min elongation at 72 °C for 50 cycles. A list of primers used in the study is given in Additional file 1: Table S1. All primers were checked for amplification efficiency to be above 90 %. Amplification efficiencies were calculated using data collected from a relative standard curve, constructed by performing serial dilutions of cDNA or purified PCR product. The relative mRNA expression was calculated using the X_0_-method, and normalized to the reference gene *GAPDH* [45]. For breast cancer samples, *HMBS* was used to control for variations in RNA concentration and integrity and was found to be the best suited reference gene when compared to *ACTB*, *GAPDH*, *YWHAZ* and *B2M* according to the Normfinder method [46]. Quantitative real-time PCR was performed using Roche LightCycler® 480 with the settings stated above. The reaction mix consisted of 5 μL SYBR Green I Master Mix Buffer (Roche), 2.5 pmol forward and reverse primers (Eurofins MWG Synthesis GmbH), 1 μL cDNA and H20 to a final volume of 10 μL. The concentration was calculated using the standard curve method. Amplicon measurements outside of the range of the standard curve, or producing an incorrect melting peak were discarded.

### Morpholino and siRNA

Morpholinos were designed by Gene Tools, LLC and transfected by the following procedure. 24 hours before transfection 5x10^4^ MDA-MB-231 cells were seeded in 12 well plates. A transfection media of 1 ml was prepared containing 6 μl Endo-Porter (6 μM), 10 μl Morpholinos (10 μM) and 984 μl DMEM growth media, added to the cells, and incubated in a CO_2_ incubator at 37 °C for 48 hours. The morpholinos had the following sequences: *STET* E2A1 ATCAGGAGAAAAAGATGATTGCCCA, *STET* E2A2 GGACTCCTTCCTATTAGTACAATGA, and Standard Control CCTCTTACCTCAGTTACAATTTATA. *STET*-targeting Morpholinos were pooled in equal amounts during preparation of transfection media. For siRNA Transfections, 100,000 MCF7 cells and 50,000 MDA-MB-231 cells were used per reaction. 20 μM siRNA stocks kept at −80 °C were diluted to 2 μM with 1x Dharmacon buffer (Thermo Scientific). 25 μl siRNA was added to 25 μl DMEM (serum and penicillin/streptomycin free) and incubated for 5 minutes. Transfection-mix was made by mixing 1 μl Dharmafect 1 (Thermo Scientific) with 49 μl DMEM (serum and penicillin free) per reaction and incubated for 5 minutes at room temperature. 50 μL siRNA was added to 50 μl transfection-mix and incubated for 20 minutes at room temperature before added to the cells following incubation in CO_2_ incubator for 72 hours. Transfections were made in duplicates for each siRNA. siRNA sequences were the following: *RRP6*, CCAGUUAUACAGACCUAU; and *RRP40*, CACGCACAGUACUAGGUC. As a negative control, Non-Targeting siRNA (Thermo Scientific, Cat. No. D-001810-10-05) was used.

### Dual reporter mini-gene constructions, transfections and genomic transpositions

Dual reporter mini-genes were constructed from the basis of the pVP4 vector, which includes a *CMV* promoter driven expression cassette with the *β − globin* exon1-intron-exon2 fused to the EGFP encoding gene [47]. In addition, pVP4 includes an expression cassette for an autonomous neomycin resistance gene. By site directed mutagenesis, an *AscI* site was inserted central in the *β − globin* intron. By *AseI* and *AscI* digestion the entire *CMV* promoter as well as the *β − globin* exon1 and 5′end of the intron was removed. A 1.1 kb PCR fragment representing the bi-directional *hnRNPA1* and *CBX5* promoter with the exon1 sequences and approximately 200 bp intron 1 sequences was inserted. The promoter fragment was inserted in two different orientations using either primers *Ase1*-*hnRNPA1*, GATCATTAATGCAAGGAACGAAACCCAGCAGCATC, and *Asc1*-*CBX5*, GATCGGCGCGCCGTCCATTCATTTCACACAATAAC or *Asc1*-*hnRNPA1*, GATCGGCGCGCCGCAAGGAACGAAACCCAGCAGCATC, and *Ase1*-*HP1α*, GATCATTAATGTCCATTCATTTCACACAATAAC and thereby generating p*CBX5*-EGFP and p*hnRNPA1*-EGFP. The vectors were cut by *AseI* and a PCR fragment inserted encompassing a 2 kb fragment with the 3′-end of the *β − globin* intron, *β − globin* exon 2, and the *katushka* reporter gene. This PCR fragment was generated with primers including *NdeI* sites, which are compatible with *AseI*. Thereby pBDf was generated that has the *katushka* transcriptional unit under control of the *hnRNPA1* promoter and the *EGFP* transcriptional unit under control of the *CBX5* promoter. pBDr has the *katushka* transcriptional unit under control of the *CBX5* promoter and the *EGFP* transcriptional unit under control of the *hnRNPA1* promoter. To generate a sleeping beauty transposon mini-gene, sbBDf, the required repetitive inverted elements were inserted to flank the *katushka* and *EGFP* transcriptional units in pBDf. A 2 kb fragment representing a continued extension of the *CBX5* intron 1 present in sbBDf was generated by PCR with primers *CBX5*-Intron1-*Asc1*-f, ACTGGGCGCGCCCGTTATTGTGTGAAATGAATG and *CBX5*-Intron1-*Asc1*-r, ACTGGGCGCGCCACTCCCTAAACATTTCAAC and cloned in the *AscI* site to generate sbBDfPE. A 2 kb PCR fragment representing the *STET* exon including 3′-flanking intron sequences and pA signal downstream sequences was generated using the primers *STET*-*Asc1*-f,

TGACGGCGCGCCAGGTTTGGTATCAGGGTACA and *STET*-*Asc1*-r, TGACGGCGCGCCATAGCAGCCACAGGAAACTA and cloned in the *AscI* sites of pBDf and sbBDf to generate pBDfS and sbBDfS, respectively. 24 hours before transfection 2x10^5^ cells were seeded in a 6 well plate. Next day, 2 μg of plasmid DNA, 6 μl X-treme gene 9 (Roche) and serum free DMEM media was mixed in a volume of 200 μl and incubated for 30 minutes at room temperature. The transfection mix was then added drop-wise to the growth media of the plated cells and incubated in CO_2_-incubator at 37 °C for 48 hours. For mini-gene genomic integration by transposition, 2x10^5^ cells were seeded in a 6 well plate the day before transfection. Next day, 2 μg of transposon mini-gene constructs, 200 ng of SB Puro and 200 ng of SB100 (10:1:1) were mixed with 7.2 μl X-treme gene 9 and serum free DMEM media in a volume of 200 μl and mixed thoroughly and incubated for 30 minutes at room temperature. The transfection mix was then added drop-wise to the growth media of the plated cells and incubated in CO_2_-incubator at 37 °C for 48 hours. The transfection media was replaced by selection media (DMEM supplemented with 1 μg/ml puromycin (Sigma)) to select for cells stably expressing the *puromycin* resistance gene. Every 2–3 days cells were washed twice with 1 ml PBS and supplied with fresh selection media.

### Chromatin immunoprecipitation (ChIP)

ChIP analyses were done essential as previously described [37, 48]. In summary, ChIP was performed with 10 ml cultures fixed with 1 % formaldehyde for 10 min followed by addition of glycine to 0.25 mM final concentration. Cross-linked cells were washed twice with cold PBS, scraped and lysed for 10 min at 4 °C in 1 % SDS, 50 mM Tris–HCl (pH 8.0) and 10 mM EDTA containing protease inhibitors. Lysates were sheared by sonication using a bioruptor (Diagenode, Liege, Belgium) to obtain chromatin fragments <0.5 kb and centrifuged for 15 min in a microfuge at 4 °C. 20 μg of soluble chromatin of each sample was incubated with antibody to the following epitopes: H3 (ab1791, Abcam, MA, USA) and H3K9ac (ab4441, Abcam) at 4 °C for 18 h and immunoprecipitated with a protein A and protein G magnetic bead mix (1:1) at 4 °C for 60 min. A mock precipitation including pre-immune polyclonal serum was included for each ChIP experiment. After sequential washing by the following buffers: three times with ChIP washing buffer I (20 mM Tris–HCl, 150 mM NaCl, 2 mM EDTA, 1 % Triton X-100. 0.1 % SDS), two times with ChIP washing buffer II (20 mM Tris–HCl, 350 mM NaCl. 2 mM EDTA, 1 % Triton X-100. 0.1 % SDS), two times with ChIP washing buffer III (20 mM Tris–HCl, 500 mM NaCl. 2 mM EDTA, 1 % Triton X-100), the chromatin was eluted from the beads with Elution buffer (100 mM NaHCO3. 1 % SDS) by rotating 15 min at room temperature. Cross-links were reversed by incubation at 65 °C for 5 to 20 h and treated with proteinase K and RNase A. DNA was purified by phenol-chloroform extraction and ethanol precipitation and eluted in 100 μl TE buffer. For quantitative detection of retained DNA, RT-qPCR were performed in triplicate and normalized to values obtained for amplicons corresponding to *GAPDH*.

### Western blot and immunofluorescence

Proteins were detected in western blotting using mouse anti-HP1α clone15.19 s2 (Millipore 05–689) in 1:1,000 dilution and rabbit anti-β − Actin (Sigma A2013) in 1:10,000 dilution. Secondary antibodies were goat anti-mouse-HRP (Dako P0447) and goat anti-rabbit-HRP (Dako P0448) in 1:10,000 dilutions. Western blot procedures using 75 μg protein extract in each lane were as previously described except using Supersignal West Dure Extended Duration Substrate (Thermo Scientific 34076) and ImageQuant LAS4000 (GE Healthcare Life Sciences) for visualization [37]. For immunofluorescence experiments cells were grown in 12 well plates on coverslips (VWR) pre-coated with Poly-L-Lysine (Sigma) to a confluence of ~60 %. Cells were cross-linked in 1 ml PBS containing formaldehyde (final concentration of 1 %) for 10 minutes at room temperature. Crosslinking was quenched by adding 114 μl 1.25 M glycine mixing by gentle pipetting in the well and incubated further for 5 minutes at room temperature. Cells were washed twice with 1 ml cold PBS and added 1 ml PBS containing 0.5 % Triton X-100 and protease inhibitors, and incubated for 15 minutes on ice. Cells were again washed twice with 1 ml cold PBS and blocked by adding 1 ml cold PBS containing 1 % BSA (Sigma) and incubated for 1 hour on ice. Primary mouse anti-HP1α antibody (1H5, Millipore) was diluted in PBS containing 1 % BSA of which 40 μl was placed on the bottom of a 10 cm^2^ petri-dish. The coverslips were placed on top of the 40 μl antibody with the cell side downwards. The petri-dish was sealed and incubated on ice for 1 hour. Coverslips were transferred to a new 12 well plate containing 1 ml cold PBS with the cell side upwards and washed 3x5 minutes on ice in 1 ml cold PBS. Secondary antibodies (Invitrogen) were diluted 1:2000 in cold PBS containing 1 % BSA, of which 1 ml was added to the coverslips after removing the PBS. The plate was wrapped in tinfoil and incubated for 30–60 minutes on ice. Coverslips were washed 5x5 minutes in 1 ml cold PBS and wrapped in tinfoil. Nuclei were dyed by adding 1 ml DAPI (Sigma) and incubating for 2–5 minutes at room temperature, and washed twice in 1 ml PBS. Coverslips were then dipped a few times in double distilled water and left to air dry in a tray wrapped in tinfoil. Coverslips were mounted on slides by adding a drop of Prolong Gold anti-fade reagent (Invitrogen) on the slide and transferring the coverslips on top with the cell side downwards.

### Statistical analysis

Statistical analyses were performed using the experimental results calculated by the X0-method from triple RT-qPCR measurements for each sample [45] or the direct relative concentrations generated from the standard curves in the patient sample experiments. *P*-values were calculated using Students paired two-tailed t-test. Each experiment was repeated minimum three times.

## Results

### HP1α down-regulation in MDA-MB-231 cells and the CBX5 and hnRNPA1 bi-directional transcriptional unit structure

**Fig. 1:**
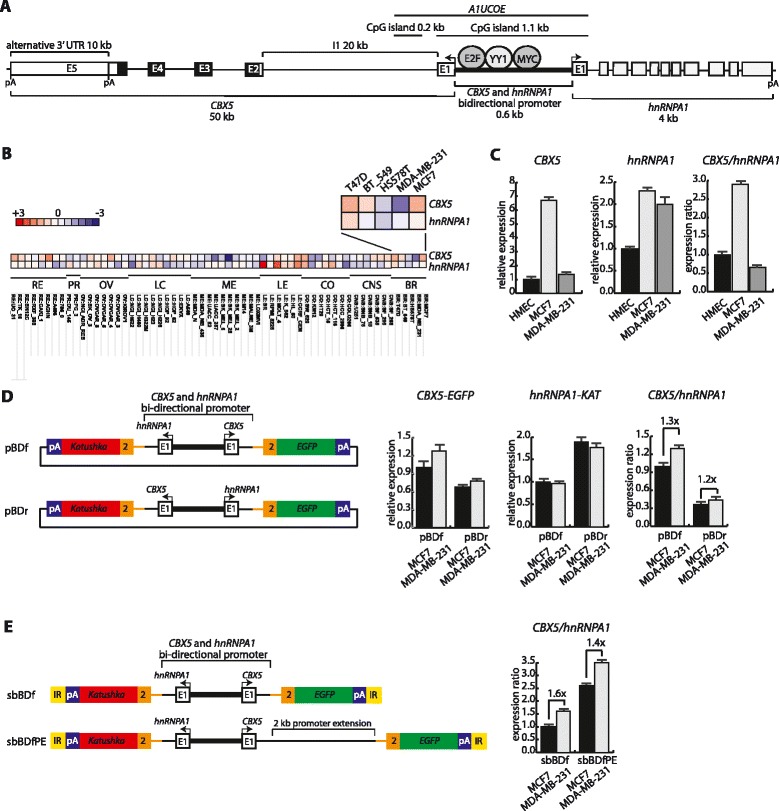
*hnRNPA1* and *CBX5* bi-directional promoter activity in breast cancer cells. **a** Schematized view of the *CBX5* and *hnRNPA1* genes (not drawn to scale). Arrows indicate direction of transcription. Localizations of transcription factor binding motifs in the bi-directional promoter were obtained from [37, 40, 41]. The HP1α coding region is indicated by black colouring. pA indicates the localization of poly-A signals. *A1UCOE* represents a CpG rich region homologous in localization to the characterized insulator element *A2UCOE* from the *hnRNPA2B1* and *CBX3* bi-directional promoter. **b** Correlation analysis of *CBX5* and *hnRNPA1* expression in the NCI-60 breast cancer cell panel. The analysis presented as heat map was performed using the CellMiner database, http://discover.nci.nih.gov/cellminer/, with red symbolizing positive and blue negative correlation. **c** Expression analysis of *CBX5* and *hnRNPA1* in HMEC, MCF7, and MDA-MB-231 cells. Relative expression was calculated from RT-qPCR using *GAPDH* expression for normalization. *CBX5* primers located to exon 4 and 5 and *hnRNPA1* primers to exon 1 and 2. **d** Transient transfection analysis of *CBX5* and *hnRNPA1* bi-directional promoter activity in dual reporter minigenes in MCF7 and MDA-MB-231 cells. 48 h after transfection RT-qPCR was used to detect relative expression levels of the spliced minigene derived reporter fusion transcripts. Expression of the vector co-expressed neomycin marker was used for normalization for transfection efficiency. Fold changes in expression ratio are shown in the right section. **e** Genomic transposition analysis of the *CBX5* and *hnRNPA1* bi-directional promoter activity in dual reporter minigenes in MCF7 and MDA-MB-231 cells. Stable cell lines generated by sleeping-beauty transposition of minigenes were analyzed by RT-qPCR to determine the expression levels of the spliced minigene derived reporter fusion transcripts. Because of copy integration number differences per transposition only the ratio of expression which was copy number independent is displayed. Fold changes in expression ratio are shown. For all panels, bars represent mean values with standard deviations

The expression of *CBX5* transcripts with coding potential for HP1α is decreased in invasive and migratory MDA-MB-231 breast cancer cells compared to the poorly invasive and migratory MCF-7 breast cancer cells [20, 36, 38, 40]. The decrease in HP1α expression is functionally linked to the enhanced invasion and migration capacity of MDA-MB-231 cells [36, 38, 40]. *CBX5* has a bi-directional promoter arrangement with *hnRNAP1* (Fig. 1a). In contrast to *CBX5*, *hnRNPA1* is relative equally expressed in MDA-MB-231 and MCF7 cells (Fig. 1, Additional file 2: Table S2 and [37]). Thus, expression regulation of cellular amounts of HP1α must mechanistically be possible without associated alterations in the housekeeping gene *hnRNPA1*. We note that previous analyses of HP1α coding mRNA regulation have been focused on the *CBX5* promoter sequences. However, due to the close proximity, it must be taken into account that an element affecting the transcriptional activity of *CBX5* could also affect the activity of *hnRNPA1*. Despite the bi-directional promoter structure, no overall significant correlation in expression pattern is observed between *CBX5* and *hnRNPA1* in the NCI-60 cancer cell line panel (correlation coefficient 0.129) (Fig. 1b and Additional file 3: Figure S1). To investigate the relation between *CBX5* and *hnRNPA1* expression, RT-qPCR analysis in HMEC, MCF7 and MDA-MB-231 cells was performed. This showed 2.9-fold up-regulation of *CBX5* relative to *hnRNPA1* in MCF7 cells versus non-cancer breast epithelial cells (HMEC) and 0.62-fold down-regulation in MDA-MB-231 cells relative to HMEC (Fig. 1c). The expression analyses supported existence of independent regulation of *CBX5* and *hnRNPA1* transcription in breast cancer cells with a concordant up-regulation of the two genes from normal cells to cancer cells and subsequently specific down-regulation of *CBX5* in metastatic cells (Fig. 1c). In a previous study, we showed that the *CBX5* promoter is less occupied by basal transcription factors such as TBP, TFIIB, TFIIH as well as Pol-II in MDA-MB-231 cells when compared to MCF7 cells [37]. The decrease in Pol-II presence was over the entire *CBX5* gene. In contrast, histone H3 and the promoter signature marks, tri-methylated lysine 4 (H3K4me3) and acetylated lysine 9 (H3K9ac) on the histone tail of H3, were equally present throughout the promoter [37]. Thus, we hypothesized two models facilitating differential regulation of *CBX5* and *hnRNPA1* from the basis of a bi-directional promoter. Either *cis*-binding of *trans*-regulators mediates specific regulation in the *CBX5* transcriptional orientation or presence of regulatory elements outside the bi-directional promoter region that control transcription specifically in the *CBX5* orientation. To test this, we constructed dual reporter mini-genes wherein the *CBX5* and *hnRNPA1* bi-directional promoter including both first exons and flanking intron sequences drives bi-directional expression of either green (EGFP) or red (Katushka) fluorescent proteins (Fig. 1d). After transient transfection into MCF7 and MDA-MB-231 cells, we observed no preferential down-regulation of *CBX5* in MDA-MB-231 cells compared to MCF7 cells (Fig. 1d). Flipping the promoter region relative to the marker genes provided similar results (Fig. 1d). Thus, we conclude that the bi-directional promoter region *per se* is not sufficient to mediate preferential *CBX5* down-regulation compared to *hnRNPA1* in MDA-MB-231 cells versus MCF7 cells.

The transient transfection approach most likely eliminates detection of putative chromatin mediated effects and can be affected by high promoter sequence copy-number mediated titration of *trans*-factors. To reduce such confounders, we constructed a sleeping beauty based transposon mini-gene with the *CBX5* and *hnRNPA1* bi-directional promoter (Fig. 1e). The mini-gene was used to generate stable genome insertion with sleeping beauty transposase in MCF7 and MDA-MB-231 cells. Pools of cells with genome insertions were examined for transcriptional orientation specific mRNA expression. The result again showed that the bi-directional promoter does not have intrinsic capacity to preferential mediate *CBX5* relative to *hnRNPA1* transcriptional down-regulation in MDA-MB-231 cells versus MCF7 cells (Fig. 1e). The mini-gene lacked complete inclusion of the two CpG islands overlapping the bi-directional promoter and we therefore generated a mini-gene with a 2 kb intron 1 extension (Fig. 1a and e). Similar to the *CBX5* and bi-directional promoter structure, *CBX3* and *hnRNPA2B1* have a 0.4 kb bi-directional promoter region, suggesting an evolutionary relationship between the HP1 encoding genes (Additional file 3: Figure S1C). The *CBX3* and *hnRNPA2B1* bi-directional transcriptional unit has been carefully analyzed due to the insulator capacity towards heterochromatin mediated gene silencing in transgenic constructs by the bi-directional promoter overlapping *A2UCOE* CpG island [49]. We note that the CpG containing fragment from the *CBX5* promoter resembles the *A2UCOE* from *CBX3* and *hnRNPA2B1*, and we abbreviate the corresponding sequence *A1UCOE*. The inclusion of *A1UCOE* had a similar positive effect on *hnRNPA1* and *CBX5* transcriptional orientations (Fig. 1e). Based on the presented expression analyses, we conclude that the observed specific down-regulation of the *CBX5* transcriptional orientation in MDA-MB-231 breast cancer cells, and thereby HP1α protein, most likely is not strictly promoter dependent, but involves promoter downstream sequences.

### Deciphering novel transcripts originating from the large intron 1 of CBX5

**Fig. 2:**
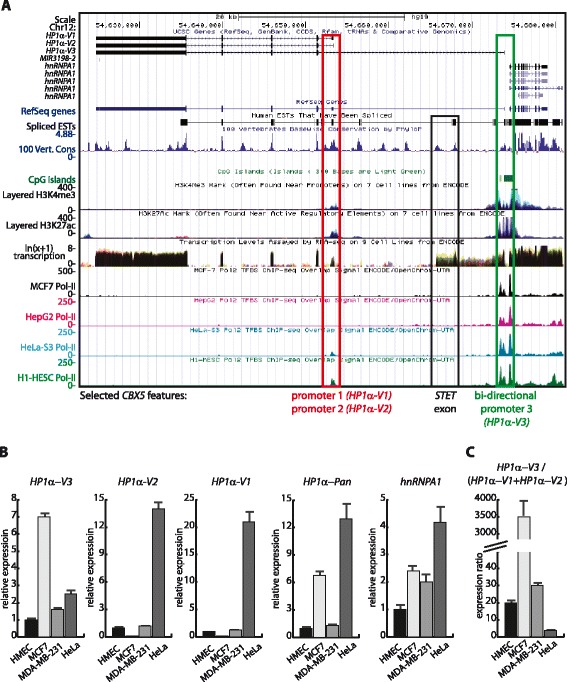
Identification of novel *CBX5* generated transcripts. **a** Screenshot from the UCSC Genome Browser showing selected features of *CBX5* and *hnRNPA1* from subtracted ENCODE datasets. On the y-axis is displayed the nature of described features. Layered H3K4me3 and H3K27ac represent ChIP-sequence results for 7 model cell lines from ENCODE. Transcription indicates the result of RNA sequencing from 9 model cell lines from ENCODE. MCF7, HePG2, HeLa-S3, and H1-HESC Pol-II indicates ChIP sequence results from the given cell lines. The numbering on the y-axix for each feature indicates the quantitative measure for the RNA and ChIP sequence results. By colored boxes and the below text is indicated the localization of the bi-directional promoter as well as intron 1 located examined alternative promoter and exon sequences. **b** Expression analysis of *CBX5* derived transcripts and *hnRNPA1* in HMEC, MCF7, MDA-MB-231, and HeLa cells. Relative expression was calculated from RT-qPCR using *GAPDH* expression for normalization. *CBX5* transcript primers were specific for the indicated isoforms and *HP1α-pan* was detected by an exon 4 and 5 primer combination. **c** Expression ratio of HP1α encoding transcripts. The ratio between *HP1α-V3* in relation to the sum of *HP1α-V1* and *HP1α-V2* was calculated based on the data in B. For all panels, bars represent mean values with standard deviations

Inspection of *CBX5* revealed existence of a large intron 1 sequence of approximately 20 kb (Fig. 1a). Intron 1 sequences are approximately 23 kb and 1 kb for *CBX1* and *CBX3* (Additional file 3: Figure S1). In an attempt to address the importance of the intron 1 sequence for *CBX5* regulation, we checked for the presence of transcriptional signatures using ENCODE data in the UCSC browser. We note that human *CBX1* and *CBX3* genes have alternative exon 1 sequences, and thereby alternative promoters (Additional file 3: Figure S1). From ENCODE derived data, two *CBX5* signatures were evident. One representing possible additional promoter sequences in the 3′-region of intron 1 and another indicating the presence of a splice form between *CBX5* exon 1 and an intron 1 embedded alternatively used exon (Fig. 2a). The latter will be described in further details below, and we will here focus on the putative alternative promoters in intron 1. Due to the existing nomenclature in UCSC of various transcriptional isoforms from *CBX5*, we will in the following term the HP1α protein-coding mRNA isoform originating from the *CBX5* and *hnRNPA1* bi-directional promoter for *HP1α-Variant 3* (*V3*). The two novel potential mRNA isoforms are termed *HP1α-Variant 1* (*V1*) and *HP1α–Variant 2* (*V2*) with the latter having the most 5′-intron 1 location of the alternative exon 1 (Fig. 2a). The ENCODE data showed peaks of promoter mark signatures, H3K27ac and H3K4me3, as well as the presence of Pol-II over the alternative exon 1 sequences for *HP1α-V1* and *HP1α-V2* (Fig. 2a). This is in support of the presence of functional promoter sequences. We note that *HP1α-V1*, *HP1α-V2*, and *HP1α-V3* mRNA isoforms all have coding potential for full-length HP1α protein given that the first consensus translational initiation codon resides in exon 2 for all three transcripts (Fig. 2a). The novel HP1α encoding transcriptional isoforms, *V1* and *V2*, could participate in generating relatively higher HP1α expression in non-metastatic MCF7 breast cancer cells without requirement of specific *CBX5* to *hnRNPA1* transcriptional enhancement from the bi-directional promoter. To address this, we performed RT-qPCR analysis specifically detecting each transcriptional isoform in HMEC, MCF7 and MDA-MB-231 cells. We observed similar expression profiles for *HP1α-V1* and *HP1α-V2. HP1α-V3* had a distinct expression profile, which was similar to HP1α encoding mRNA detected by primers located in exons 4 and 5 and thereby the three isoforms altogether (*HP1α-pan*) (Fig. 2b). PCR experiments showed that the *HP1α-V3* expression ratio relative to *HP1α-V1* and *HP1α-V2* was approximately 20-fold higher in HMEC, 3,500-fold higher in MCF7 and 30-fold higher in MDA-MB-231 c**e**lls (Fig. 2c). Thus, expression data did not support that *HP1α-V1* and *HP1α-V2* transcripts contribute significantly to the overall HP1α encoding transcript levels in neither MCF7 nor MDA-MB-231 cells. ENCODE data showed the highest peak of Pol-II over the alternative promoter sequences in HeLa cells (Fig. 2a). By RT-qPCR, we also found that HeLa cells express *HP1α-V1* and *HP1α-V2* at a level comparable to *HP1α-V3* (Fig. 2b and c). Thus, the HP1α encoding *V1* and *V2* transcripts might in some cellular contexts quantitatively contribute to the total HP1α encoding transcript levels. In conclusion, the analysis of the novel *HP1α-V1* and *HP1α-V2* transcript isoforms were not supportive for a role directly involved in HP1α transcript and protein down-regulation in MDA-MB-231 compared to MCF7 cells.

**Fig. 3:**
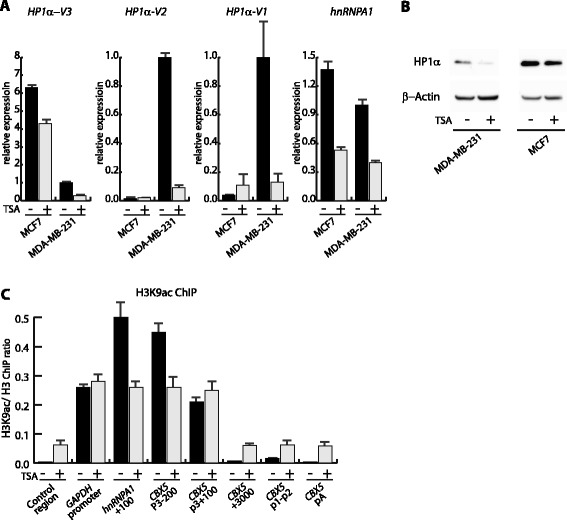
TSA is a negative regulator of the *CBX5* and *hnRNPA1* locus. **a** TSA mediates *CBX5* down-regulation. Relative expression of *CBX5* derived transcripts and *hnRNPA1* in MCF7 and MDA-MB-231 cells in a TSA response after 24 h treatment. Relative expression was calculated from RT-qPCR using *GAPDH* expression for normalization. *CBX5* transcript primers were specific for the indicated isoforms and *HP1α-pan* was detected by an exon 4 and 5 primer combination. **b** TSA down-regulates HP1α expression at the protein level. Protein extracts were isolated from MCF7 and MDA-MB-231 cells in a TSA response after 24 h treatment. Western blot analyses were performed with antibodies for HP1α and β − Actin for loading control using the same membrane. **c** H3-K9ac and H3 ChIP analysis of the *CBX5*-*hnRNPA1* locus. ChIP analysis were performed from MDA-MB-231 control cells or treated with TSA for 24 h. The presented data shows the H3K9ac signal relative to H3 signal. Positions of PCR primers are indicated. The control region is located in the un-transcribed genomic position. For all panels, bars represent mean values with standard deviations

We have previously shown that the H3 content over *CBX5* is equal in MCF7 and MDA-MB-231 cells, whereas the chromatin mark coupled with transcriptional elongation, H3K36me3, was decreased over the *CBX5* gene body in MDA-MB-231 cells compared to MCF7 cells [37]. Chromatin compaction in *CBX5* intron 1 could contribute to the low expression of *HP1α-V1* and *HP1α-V2*. To address chromatin-mediated regulation, we treated MCF7 and MDA-MB-231 cells with the histone de-acetylase inhibitor trichostatin-A (TSA). Previous results have shown equal amounts of H3K9ac at the *CBX5* promoter in MCF7 and MDA-MB-231 cells [37]. To our surprise, we observed that in MDA-MB-231 cells TSA treatment resulted in 5-fold decreased *CBX5* expression for all three HP1α encoding transcript isoforms (Fig. 3a). In MCF7, no significant TSA effect was observed (Fig. 3a). *hnRNPA1* expression was 2-fold decreased following TSA treatment and this was also observed in MCF7 cells (Fig. 3a). We also observed HP1α protein down-regulation by western blotting and immunofluorescence analysis (Fig. 3b and Additional file 4: Figure S2B and S2C). ChIP analysis showed that the H3K9ac/H3 ratio in MDA-MB-231 cells decreased or was equal at the *CBX5* and *hnRNPA1* bi-directional promoter and increased at *CBX5* downstream sequences following TSA treatment (Fig. 3c). Notably, the ChIP results for the alternative promoter regions for *HP1α-V1* and *HP1α-V2* showed a 3-fold increased level of H3K9ac, which did not correlate with increased mRNA expression (Fig. 3c).

### Identifying a novel transcript isoform, STET, originating from alternative splicing and polyadenylation in intron 1 of CBX5

**Fig. 4:**
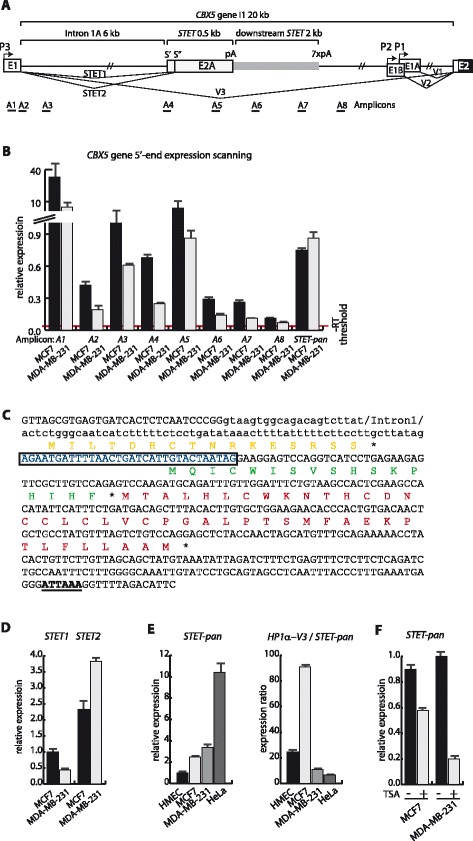
*CBX5* intron 1 generates a novel transcript *STET*. **a** Schematized view of *CBX5* intron 1. Arrows indicate direction of transcription and rectangles indicate exon sequences. The HP1α coding region is indicated by black colouring. pA indicates the localization of poly-A signals. *E1* is the first exon for *HP1α-V3*, *E1A* is the first exon for *HP1α-V1*, *E1B* is the first exon for *HP1α-V2*, and *E2A* the terminal composite exon for the *STET* transcript. The two different *STET* transcripts, *STET1* and *STET2*, using different splice sites are also indicated. Below the intron drawing is indicated location of amplicons used to detect transcription throughout the *CBX5* intron 1. **b** Scanning of *CBX5* intron 1 5'-region for expressed transcripts. Primer localizations for the various amplicons is shown in A. *STET-pan* indicates the measurement of the spliced *STET* transcript. Relative expression was calculated from RT-qPCR using *GAPDH* expression for normalization. –RT threshold line in red shows the level of background calculated from paired samples with a cDNA syntheses reaction without reverse transcriptase to account for the detection of contaminating DNA. **c** Nucleotide sequence of the *STET E2A* and surrounding sequences. The exon sequence extension in *STET1* is enclosed by rectangle. pA signal is underlined and in bold. Different ORF’s are shown with the peptide sequences in color. **d** Expression analysis of *STET* transcripts in MCF7 and MDA-MB-231 cells. Relative expression was calculated from RT-qPCR using *GAPDH* expression for normalization. **e** Expression analysis of *STET-pan* in HMEC, MCF7, MDA-MB-231, and HeLa cells. Relative expression was calculated from RT-qPCR using *GAPDH* expression for normalization. The calculated semi-quantitative expression ratio between *HP1α-V3* and *STET* is shown to the right. **f** TSA mediates *STET* transcript down-regulation. Relative expression of *STET-pan* mRNA in MCF7 and MDA-MB-231 cells after 24 h TSA or DMSO control treatment. Relative expression was calculated from RT-qPCR using *GAPDH* expression for normalization. For all panels, bars represent mean values with standard deviations

To further delineate the transcriptional structure of *CBX5* intron 1 we next focused on an embedded alternative exon indicated by transcriptional signatures using ENCODE data in the UCSC browser. *In silico* a *CBX5* transcript was identified consisting of exon 1 fused to this alternative spliced and polyadenylated exon embedded in intron 1 (Figs. 2a and 4a). We abbreviated this transcript for *CBX5* skipped terminal exon transcript (*STET*). To verify the expression of *STET*, and eventual other *CBX5* intron 1 derived transcripts, we screened for RNA expression using RT-PCR amplicons representing different intron 1 positions (Fig. 4a). Relative to amplicon *A4* representing the intron 1 to *STET1* exon boundary we observed an increase in RNA levels particularly in MDA-MB-231 cells corresponding to amplicon *A5* representing the *STET* 3′-UTR (Fig. 4b). Amplicons located further downstream in intron 1 showed pronounced decrease in RNA levels in accordance with a major transcriptional stop mediated by the *STET* pA signal (Fig. 4b). We notice the presence of an array of 7 consensus pA signals 2 kb downstream from the *STET* pA signal in the *CBX5* intron 1 sequence. Downstream of this multiple pA signal array, RNA transcript levels approached background (Fig. 4b). However, we could not identify transcripts terminated by the seven downstream pA signals by 3′-RACE. We note that downstream AU-rich regions can be important contributors in co-transcriptional cleavage (CoTC). During CoTC cleavage of the nascent transcript occurs 1–2 kb downstream of the polyadenylation signaling event, thereby releasing the polymerase followed by a subsequent cleavage at the pA signal [50]. In conclusion, the expression data supported the existence of significant amounts of cellular RNA representing intron 1, including the STET exon (Fig. 4b). Further RT-PCR analyses and sequencing of amplicons verified the existence of *STET* mRNA in both MCF7 and MDA-MB-231 cells and that two different *STET* mRNAs were present (Fig. 4a-d). *STET1* included an additional extension of 32 bases in the 5′-end compared to *STET2* (Fig. 4a and c). 3′-RACE analyses showed that the *STET* isoforms were polyadenylated from an AUUAAA pA signal resulting in exon lengths of 383 and 351 bp for *STET1* and *STET2*, respectively (Fig. 4c). Of additional sequence elements required for a functional pA signal, we note the presence of an upstream UGUA-element and downstream U-rich elements, which mediates binding of CstF-64 [51] surrounding the *STET* AUUAAA motif (Fig. 4c). BLAST searches identified significant *STET* evolutionary conservation in various primates, including existence of the two alternative splice forms of *STET* in e.g. marmoset, but absence of *STET* in rodents. PCR experiments showed that *STET2* mRNA was more abundant than *STET1* mRNA in MCF7 and MDA-MB-231 cells (Fig. 4d). However, whereas *STET1* was 0.43-fold down-regulated in MDA-MB-231 compared to MCF7 cells, *STET2* was 1.65-fold up-regulated (Fig. 4d). Using a primer set detecting both *STET* mRNA isoforms, STET-pan, we observed that *HP1α-V3* down-regulation in MDA-MB-231 versus MCF7 cells was not linked to decreased STET expression supporting independent regulation of the transcript levels (Fig. 4e). In contrast, TSA treatment resulted in similar response profiles for *STET* and *HP1α-V3* (Figs. 4f and 3a).

### Increased STET mRNA expression is not directly functionally associated with down-regulation of HP1α encoding mRNA

Each coupled alternative splicing and pA event resulting in one *STET* mRNA could decrease the generation of one consensus HP1α encoding transcript isoform *V3* through *STET* exon pA mediated transcriptional termination. In a straightforward hypothesis, the increased generation of *STET* mRNA could mediate HP1α encoding mRNA down-regulation in metastatic breast cancer cells. In this case, the generation of *STET* transcripts would be at the same order of magnitude as HP1α encoding transcripts and *STET* mRNA down-regulation would follow *HP1α-V3* mRNA up-regulation and *vice versa*. Expression analysis, however, showed a *HP1α-V3* relative to *STET* expression in the order of 90-fold in MCF cells and 10-fold in MDA-MB-231 cells (Fig. 4e). *HP1α-V3* mRNA is stable and low *STET* mRNA stability could lead to underestimation of the *STET* mRNA synthesis rate [37, 41]. This was not the case as RNA stability analysis showed an approximately similar stability of *STET* mRNA and *HP1α-V3* mRNA (Additional file 5: Figure S3A). Moreover, rapid degradation of nascent *STET* mRNA by the RNA exosome could decrease the steady-state levels. siRNA mediated depletion of essential exosome components RRP6 and RRP40 influenced *HP1α-V3* mRNA and *STET* mRNA levels, but not in an order of magnitude to support this mechanism to be involved generating preferential low levels of *STET* mRNA (Additional file 5: Figure S3B and C).

**Fig. 5:**
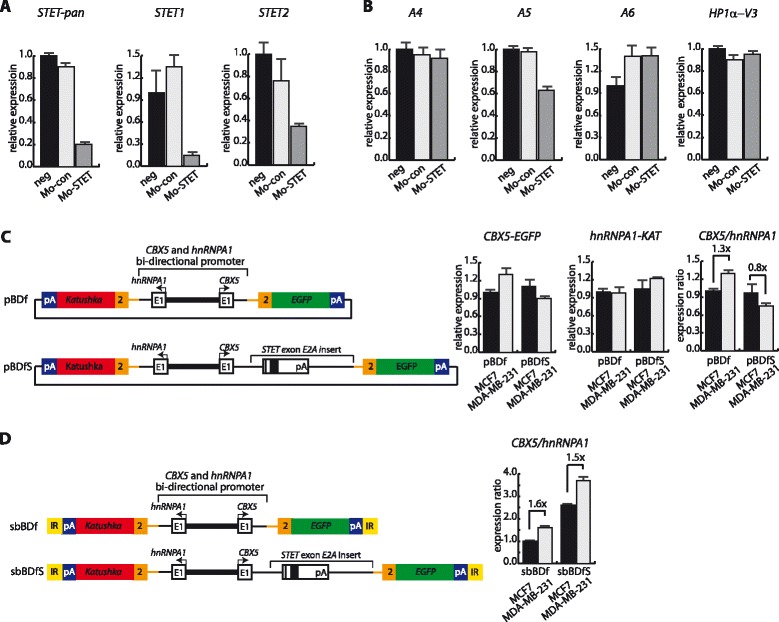
Characterization of *STET* in *CBX5* regulation. **a** Down-regulation of *STET* mRNA levels by morpholino transfection. MDA-MB-231 cells were transfected with morpholino’s directed against the *STET* intron and exon boundary or control morpholino. Relative expression was calculated using RT-qPCR using *GAPDH* expression for normalization. Neg, mock transfected cells; Mo-con, morpholino control transfected cells; and Mo-STET, *STET*-specific morpholino transfected cells. **b** *STET* morpholino transfection has no significant effect on *HP1α-V3* expression. MDA-MB-231 cells were transfected with morpholino’s as described in A. *A4*, *A5*, and *A6* represents amplicons in *CBX5* intron 1 described in Fig. 4. Relative expression was calculated using RT-qPCR using *GAPDH* expression for normalization. **c** Transient transfection analysis of *CBX5* and *hnRNPA1* bi-directional promoter activity without and with *STET* exon insert in dual reporter minigenes in MCF7 and MDA-MB-231 cells. 48 h after transfection RT-qPCR was used to detect relative expression levels of the spliced minigene derived reporter fusion transcripts. Expression of the vector co-expressed neomycin marker was used for normalization for transfection efficiency. Fold changes in expression ratio are shown in the right section. **d** Genomic transposition analysis of the *CBX5* and *hnRNPA1* bi-directional promoter activity without and with *STET* exon insert in dual reporter minigenes in MCF7 and MDA-MB-231 cells. Stable cell lines generated by sleeping-beauty transposition of minigenes were analyzed by RT-qPCR to determine the expression levels of the spliced minigene derived reporter fusion transcripts. Because of copy integration number differences per transposition only the ratio of expression which was copy number independent is displayed. Fold changes in expression ratio are shown. For all panels, bars represent mean values with standard deviations

We next examined if down-regulation of *STET* mRNA directly associated with *HP1α-V3* mRNA up-regulation. For this, we examined the consequences of down-regulating *STET* synthesis with morpholinos corresponding to the splice sites for the *STET* exon. Morpholino transfection of MDA-MB-231 cells with an equal mix of morpholinos targeting either of the splice sites resulted in 5-fold decrease in the amounts of spliced *STET* transcripts (Fig. 5a). This was not accompanied by a similar alteration in the amounts of un-spliced *STET* mRNA (amplicon A4) or RNA corresponding to the downstream utilized *STET* pA signal (amplicon A6) (Fig. 5a). A 0.63-fold decrease in expression was observed using an amplicon detecting both spliced and un-spliced *STET* (amplicon *A5*) (Fig. 5b). This was in accordance with a linkage between blocking of *STET* mRNA splicing and blocking of *STET* pA signal usage altogether resulting in a decrease in total *STET* generation. No corresponding increase in *HP1α-V3* mRNA expression was observed un-favouring that *STET* mRNA generation is directly linked with *HP1α-V3* mRNA generation in a significant amount (Fig. 5b). This cannot rule out the possibility for a stoichiometric effect such that each *STET* mRNA generated is accompanied with a decreased generation of one *HP1α-V3* mRNA, but since the total amount of *STET* mRNA relative to *HP1α-V3* mRNA is low this will be left undetectable. Finally, we examined how insertion of the *STET* exon in a dual reporter mini-gene under transcriptional control of the *hnRNPA1* and *CBX5* bi-directional promoter influenced expression. For this, we used the same expression vectors as described in Fig. 1 with the addition of a 1.17 kb *STET* exon and flanking sequences insert (Fig. 5c). Transient transfections in MCF7 and MDA-MB-231 cells resulted in no significant decrease in the *CBX5* transcriptional orientation by inclusion of the *STET* exon (Fig. 5c). In cell lines with mini-gene integrations by transposition, we also did not detect significant *STET* exon mediated effects on the *CBX5* transcriptional orientation (Fig. 5d). In conclusion, data did not support a model wherein *STET* exon sequences are mechanistically involved in abolishing the inclusion of downstream consensus HP1α encoding exons in quantitative amounts to mediate significant down-regulation of *HP1α-V3* mRNA expression. Instead, the results favour that transcriptional down-regulation of the *CBX5* gene in MDA-MB-231 cells increases the relative abundance of coupled *STET* exon alternative splicing and polyadenylation with a resulting increase in *STET* mRNA expression.

### hnRNAP1, HP1α-V3 and STET mRNA expression during breast cancer progression

**Fig. 6:**
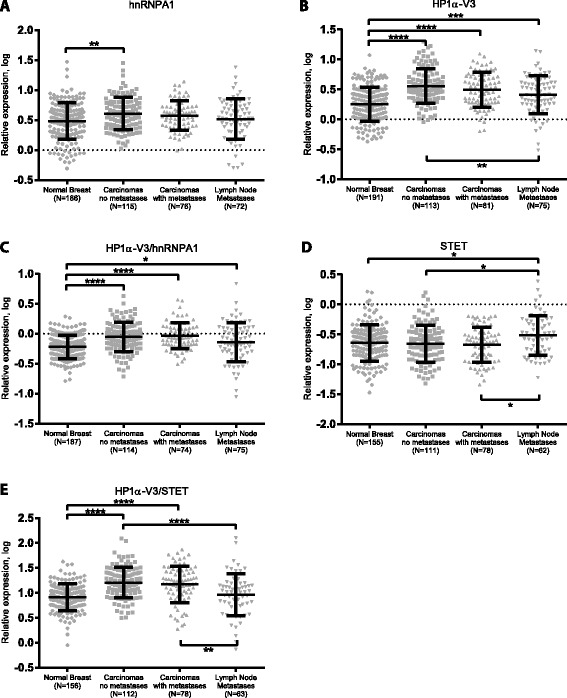
Expression analysis of *hnRNPA1*, *HP1α-V3* and *STET* in breast cancer biopsies. **a**-**e** Based on a standard curve of serial dilutions of cDNA with known concentrations, quantification was determined from single measurements with the second derivate max method by the LightCycler software. Relative expression was calculated using *HMBS* for normalization. Results are presented as log-transformed values of *HMBS* normalized data. N indicates the number of samples with measurements above limit of detection. The bars represent the mean value with surrounding standard deviations. * *P* < 0.05; ** *P* < 0.01; *** *P* < 0.001; **** *P* < 0.0001. One-way ANOVA with Tukey’s multiple comparisons. For datasets with significantly different standard deviations between means (*HP1α-V3*/*hnRNPA1* and *HP1α-V3*/*STET*), non-parametric Kruskal-Wallis tests with Dunn’s multiple comparisons were performed

We next analysed the relationship between *hnRNPA1*, *HP1α-V3* and *STET* mRNA expression in breast cancer samples to see whether this corresponds to observations from breast cancer cell lines. cDNA was prepared from paired tissue samples from 193 patients with breast cancer [43, 44]. 81 of the patients had metastases in the lymph nodes. From each patient a sample of normal breast tissue and primary breast carcinoma were obtained. From 78 of the patients a sample from the lymph node metastases was obtained. Expression levels for *HP1α-V3*, *STET*, and *hnRNPA1* mRNA were measured by RT-qPCR in the normal breast, primary carcinoma samples and lymph node metastases. To acquire a normal distribution, the normalized expression values were log-transformed and all datasets passed the D’Agastino-Pearson normality test. Compared to normal breast tissue samples, *HP1α-V3* mRNA expression was higher in both primary carcinoma samples from patients with lymph node metastasis (1.75-fold, *P* < 0.0001) and without lymph metastasis (2.02-fold, *P* < 0.0001) (Fig. 6b). *HP1α-V3* expression was also higher in lymph node metastases compared to normal breast tissue (1.44-fold, *P* < 0.001) (Fig. 6b). *HP1α-V3* expression in the primary carcinoma samples from patients without lymph node metastases was significantly higher than expression in lymph node metastases samples (1.40-fold, *P* < 0.01) (Fig. 6b). Albeit not statistically significant, there was also a tendency towards down-regulation of *HP1α-V3* expression in primary carcinoma from patients with metastases compared to lymph nodes metastases (Fig. 6b). For *hnRNPA1* mRNA we observed an increased expression between normal breast tissue and primary carcinoma samples from patients without metastasis (1.33-fold, *P* < 0.01) (Fig. 6a). *hnRNPA1* also appeared upregulated in primary carcinoma samples from patients with metastasis, but not significantly (Fig. 6a). No difference in *hnRNPA1* mRNA expression was observed between primary carcinoma and lymph node metastases samples (Fig. 6a). These results support the HMEC, MCF7 and MDA-MB-231 cell line results. For the expression ratio between *HP1α-V3* and *hnRNPA1*, we observed an increase between normal breast tissue and primary carcinoma samples both from patients without metastasis (1.46-fold, *P* < 0.0001) and patients with metastasis (1.79-fold, *P* < 0.0001) (Fig. 6c). Also an increase from normal breast tissue to lymph node metastases was significant (1.2-fold, *P* < 0.05) (Fig. 6c). Thereby, the results from the *in vivo* material were in agreement with the results from cancer cell lines. No significant difference in *STET* mRNA expression was found between normal tissue and primary carcinoma from patients with or without lymph node metastasis (Fig. 6d). However, expression of *STET* was in normal breast lower than in lymph node metastases (0.75-fold, *P* < 0.05) (Fig. 6d). *STET* expression was also lower in carcinomas from patients without metastasis (0.73-fold, *P* < 0.05) and with metastasis (0.70-fold, *P* < 0.05) relative to lymph node metastases (Fig. 6d). The *HP1α-V3* to *STET* ratio was significantly higher in primary carcinomas both from patients without metastasis (1.97-fold, *P* < 0.0001) and with metastasis (1.81-fold, *P* < 0.0001) relative to normal breast (Fig. 6e). The *HP1α-V3* to *STET* ratio was also significantly higher in primary carcinomas both from patients without metastasis (1.76-fold, *P* < 0.0001) and with metastasis (1.61-fold, *P* < 0.01), compared to lymph node metastases (Fig. 6e). To account for variation in baseline *HP1α-V3* mRNA expression levels between patients, we performed an alternative data analysis where we normalized the expression of *HP1α-V3* and *STET* mRNA to the corresponding normal breast tissue sample (Additional file 6: Figure S4). For the *HP1α-V3* to *STET* mRNA expression ratio, significant differences were observed for both primary carcinoma samples both from patients with metastasis (1.81-fold, *P* < 0.01) and without metastasis (1.68-fold, *P* < 0.01) compared to lymph node metastases (Additional file 6: Figure S4). Thus, irrespective of our data analysis method, we identified inverse correlation between *HP1α-V3* and *STET* mRNA expression levels for primary breast carcinoma versus lymph node metastases. These findings are in alignment with the data presented for HMEC, MCF7 and MDA-MB-231 cell lines.

## Discussion

HP1α is over-expressed in several types of cancers and the over-expression is associated with increased cell proliferation most likely through silencing of cell proliferation inhibitors [34]. Moreover, HP1α has a proliferation dependent expression level, which is reduced under transient cell cycle exit [34]. Finally, a decrease in HP1α expression is functionally associated with an increased invasive potential of breast cancer cells most likely due to decreased silencing of pro-invasive genes. The Janus-faced regulation of HP1α expression during carcinogenesis can be a reflection of the inverse correlation that has been suggested between cancer cell proliferation and invasion [34, 52]. Cancer cell metastasis requires an acquisition of invasive potential and adaptation to a new environment, which can be incompatible with a high proliferation rate. A temporal slowdown of cell proliferation, accompanied by down-regulation of HP1α expression, can permit the expression of pro-invasive genes, thereby resulting in metastasis [34]. However, the outgrowth of metastases requires cell proliferation and this process can be paramount for the final patient outcome, since high HP1α expression correlates with earlier diagnosis of metastasis [34]. Because of the inverse correlation between HP1α expression and the invasive potential of cancer cells, knowledge on the differential regulation of HP1α expression is an important prospect in fundamental cancer research [34]. In this study, we have shown that the decrease in HP1α expression in metastatic breast cancer cells involves promoter downstream sequences of the HP1α encoding *CBX5* gene. *hnRNPA1* and *CBX5* shares a bi-directional promoter structure. Such “head-to-head” gene arrangements are found at a high frequency throughout the human genome with ~11 % of all genes defined as bi-directional promoter genes by being divergently transcribed, and with transcriptional start sites (TSS) less than 1 kb away from each other [53–55]. Bi-directional arrangements are often evolutionary conserved, indicating functional importance of this specific gene structural modulation [56–58]. In this line, expression of bi-directional promoter genes are more correlated than those of randomly selected and neighboring genes. This is exemplified by gene pairs needed in stoichiometric amounts e.g. histone genes, functioning in the same biological pathway e.g. DNA repair, and co-expressed at specific time points during cycle or in response to induction signals e.g. heat shock [54, 59]. Bi-directional promoter sequences share several features separating them from the general non-bi-directional promoters [58]. Bi-directional promoters are more often located within a CpG island (77 %) compared to non-bi-directional promoters (38 %) and bi-directional promoters have a GC-content (66 %), which is higher than non-bi-directional promoters (53 %) [56–58]. Furthermore, the relative presence of canonical TATA box elements is significantly less for bi-directional promoters (8 %) compared to other promoters (28 %) [54, 60]. Bi-directional promoters display an enriched occurrence of specific transcription factor binding sites, including GABPA, MYC, E2F, NRF and YY1 [59]. These *cis*-elements are often functionally shared for both transcriptional directions [54, 61]. The *hnRNPA1* and *CBX5* bi-directional promoter lacks TATA box elements, contains a CpG island, and includes binding motifs for signature bi-directional promoter transcription factors e.g. YY1, E2F and MYC, and thus is a consensus representative of bi-directional promoters [37, 40, 41, 62, 63]. We identified no significant correlated expression pattern between *hnRNPA1* and *CBX5* in the NCI-60 cancer cell panel. This is illustrated by the *CBX5* down-regulation in metastatic breast cancer cells compared to poorly invasive breast cancer cell lines whereas *hnRNPA1* is relatively evenly expressed in both types of cell lines [36, 37, 40, 41]. However, we note that from normal breast epithelial cells to MCF7 cells both *hnRNPA1* and *CBX5* are up-regulated. The scenario is different for the *CBX3* and *hnRNPA2B1* bi-directional promoter that results in a highly correlated expression of these two genes in the NCI-60 cancer cell panel including MCF7 and MDA-MB-231 cells. Thus, for *hnRNPA1* and *CBX5* evolution must have adapted regulatory mechanisms un-coupling the expression of the two genes under certain cellular environments e.g. during breast cancer metastasis. Going from an *in vitro* breast cancer cell model using HMEC, MCF7 and MDA-MB-231 cells to clinical breast cancer samples, we could largely replicate findings concerning the up-regulation of both *hnRNPA1* and *CBX5* in carcinoma versus normal breast epithelial cells, de-coupling of *hnRNAP1* and *CBX5* expression in metastatic breast cancer cells, and the relative up-regulation of the *STET* transcript in metastatic breast cancer cells. This validates that in this case our cell line based model is valuable for investigating *in vivo* breast cancer progression.

Previous studies have focused on human *CBX5* regulation in terms of *cis*-elements and transcription factor binding to the consensus promoter region upstream and in exon 1. This resulted in the identification of a USF/C-MYC recognition site upstream for the *CBX5* transcriptional start site to be involved mediating differential expression in invasive versus poorly invasive breast cancer cells [40]. Based on observations in both transient and genome integrated reporter systems, our presented analyses point to importance of also promoter downstream transcriptional regulatory events. We observed that the isolated *hnRNPA1* and *CBX5* bi-directional promoter shows no significant preference for *CBX5* relative to *hnRNPA1* down-regulation in MDA-MB-231 cells. One hint of a transcriptional regulatory mechanism for *CBX5* beyond promoter mediated initiation comes from a recent study of transcriptional pausing [64]. The majority of human genes are at the promoter proximal region loaded with paused Pol-II poised for release by the positive elongation factor pTEFb into productive elongation. Gdown1 was shown to be a sub-stoichiometric subunit of Pol-II complex. Gdown1 inhibits termination of Pol II by TTF2 thereby preventing release of short transcripts and Pol-II dissociation, blocking elongation stimulation by TFIIF and influencing pausing factors NEFL and DSIF. The *hnRNPA1* and *CBX5* promoters are both associated with Gdown1 and poised Pol-II [64]. Notably, two such Pol-II and Gdown1 peaks are present at *CBX5* 50 and 450 bp downstream of the bi-directional promoter [64]. Since binding of Gdown1 to the promoter is linked with efficient transcriptional elongation and promoting stability of the paused Pol-II complex, deficiency in Gdown1 functional association to the bi-directional promoter in MDA-MB-231 cells could be a theoretical possibility. Thus, a skewed expression pattern in favor of *hnRNPA1* expression relative to *CBX5* expression will be obtainable, given the Gdown1 effect is directed specifically towards *CBX5* in a yet not proven mechanism. This scenario is in line with our previous observation of less abundance of the transcriptional elongation chromatin mark H3K36me3 in MDA-MB-231 cells [37, 65]. Importantly, we observed that throughout the *CBX5* gene, with the exception of the *STET* transcript to be discussed below, lesser amounts of transcripts were present in MDA-MB-231 cells compared to MCF7 cells. We have described that in MDA-MB-231 cells less Pol-II was present at the promoter compared to MCF7 cells [37]. We note that Gdown1 is equally expressed in MCF7 and MDA-MB-231 cells (Additional file 2: Table S2).

Another possibility in breast cancer cells to mechanistically disconnect generation of HP1α from the functionally shared promoter architecture for *CBX5* and *hnRNPA1* is the use of downstream alternative promoters producing HP1α coding transcripts. We identified two such alternative promoters in the *CBX5* intron 1 resulting in two additional HP1α encoding transcripts, *HP1α-V1* and *HP1α-V2*. Both transcripts contain the entire full-length HP1α coding region, but with an alternative first exon not included in the canonical HP1α encoding transcript, *HP1α-V3*. However, in the cancer cell lines the quantitative significant production of HP1α was concluded to be restricted to transcripts produced from the bi-directional promoter. In HMEC, MCF7 and MDA-MB-231 cells we observed an inverse expression pattern of *HP1α-V3* compared to *HP1α-V1* and *HP1α-V2*. This could be a consequence of transcriptional interference where high transcriptional rate dictated from the bi-directional promoter repressed activity of the downstream alternative promoters. It should not be ruled out that the alternative *CBX5* promoters e.g. in certain cell or tissue types or during cell cycle or developmental stages, could contribute significantly to HP1α expression. In line with this, we note that we in HeLa cells observed a significant contribution of theses alternative transcripts to the total content of HP1α encoding mRNA. Recently, an alternative downstream promoter for generating HP1α encoding mRNA was described in mice and the significance can be based upon a high degree of sequence conservation in mammalians of the genomic region corresponding to the alternative promoters [66]. Surprisingly, we observed after TSA treatment of MDA-MB-231 cells a coordinated and strong down-regulation of *HP1α-V3*, *HP1α-V1*, *HP1α-V2,* and *STET* transcripts as well as down-regulation of HP1α protein expression. *hnRNPA1* was also down-regulated but to a lesser extent. In MCF7 cells *hnRNPA1* was repressed by TSA in magnitude similar to MDA-MB-231 cells whereas *CBX5* transcripts displayed only a minor and non-coordinated response. MDA-MB-231 ChIP experiments showed no TSA induced increase in levels of acetylated histone H3 at the bi-directional promoter region. Repression of the *CBX5-hnRNPA1* locus could be mediated through recruitment of a TSA induced *trans*-repressor. However, we note that the TSA induced decrease of the *CBX5* transcripts in MDA-MB-231 cells was more pronounced than could expected for only an effect on transcription given the relative high mRNA stability (Additional file 5: Figure S3 and [37]). This could indicate TSA induced de-stabilization of *CBX5* transcripts, similar to e.g. *claudin-1* mRNA [67].

Bioinformatics analysis, as well as analysis of the mouse *CBX5* gene, revealed presence of several evolutionary conserved regions and transcribed regions in *CBX5* intron 1 [66]. Our further investigation of one such region led to the identification of two novel transcripts from *CBX5* termed *STET1* and *STET2*. These are transcribed from the same promoter as *HP1α-V3* mRNA and thereby contain exon 1 which is now spliced to an intron 1 embedded alternative exon located ~5 kb downstream of the TSS. The alternative *STET* exon includes a functional pA signal. *STET* mRNA generation thereby constitute an alternative cleavage and polyadenylation (APA) event and the *STET* exon E2A classifies as a composite terminal exon. Studies analyzing APA events have mainly been focused on the 3′-UTR, but RNA-sequence analysis have revealed that 20 % of human genes have at least one intronic APA event and that the APA events can be developmental and cell cycle regulated to regulate expression [68–70]. We note that E2F transcription factors were described to enhance alternative intronic polyadenylation in a cell proliferative dependent manner and the presence of a E2F *cis*-element in the bi-directional promoter could provide a link to *STET* mRNA generation [71]. Mechanistic selection of *STET* alternative splicing and polyadenylation is expected in stoichiometric amounts to decrease the generation of *HP1α-V3* transcripts with HP1α encoding potential. Given that *STET* is relatively more expressed in MDA-MB-231 cells compared to MCF7 cells this opens an appealing model for the specific *CBX5* relative to *hnRNPA1* down-regulation in MDA-MB-231 cells. This will however require that *STET* mRNA generation in quantitative amounts is comparable with HP1α encoding mRNA. Since our analyses systematically identified *STET* mRNA in minor amounts compared to HP1α encoding mRNA, we have no supportive evidence for such a regulatory model. Furthermore, insertion of the *STET* composite terminal exon in a mini-gene background had neither in transient nor genome integrated analysis a negative influence for the inclusion of a *STET* exon downstream located exon. Finally, despite that we find the bi-directional promoter equally transcriptional prone in MCF7 and MDA-MB-231 cells, we also observed less exon 1 included transcripts and less Pol-II, TBP, TFIIB, and TFIIH loading on the canonical *CBX5* promoter in MDA-MB-231 cells indicating less transcription [37]*.* It is important to notice that histone H3 as well as H3K9ac and H3K4me3 occupancy over the *CBX5* promoter was similar in the two cell lines pointing that the chromatin structure *per se* is not inhibitory in MDA-MB-231 cells [37]. Thereby, the current results could fit a model wherein reduced *CBX5* transcriptional quality in metastatic breast cancer cells mediated by downstream elements e.g. through impaired transcriptional re-initiation and elongation, results in relative increased inclusion of the *STET* composite exon.

History has dictated genes to be perceived as linear entities confined by promoters and terminators that determine where transcription starts and ends. Studies concerning the regulation of HP1α have hence mainly been restricted to the canonical *CBX5* promoter region. However, our presented results for the differentially expressed *CBX5* mRNA and the constitutively expressed *hnRNPA1* mRNA have indicated novel mechanisms associated with regulation of HP1α expression through sequences located downstream the bi-directional promoter. The present study highlights the need for additional focus on the transcriptional regulatory mechanistic backgrounds for deregulated HP1α expression under development and metastatic progression of breast cancer.

## Conclusion

In this study, we demonstrate that an *hnRNPA1* and *CBX5* bi-directional core promoter fragment shows no significant preference for *CBX5* relative to *hnRNPA1* down-regulation in metastatic MDA-MB-231 cells. Thus, we conclude that the bi-directional promoter region *per se* is not sufficient to mediate preferential *CBX5* down-regulation compared to *hnRNPA1* in MDA-MB-231 cells versus MCF7 cells, but involve sequences located downstream the canonical *CBX5* promoter. Characterization of transcriptional events in the *CBX5* 20 kb long intron 1 revealed existence of several novel *CBX5* transcripts. Two of these encoded consensus HP1α protein but used autonomous promoters located within intron 1 by which HP1α expression could be de-coupled from the bi-directional promoter. However, in breast cancer cell lines a quantitative significant production of HP1α was concluded to be restricted to transcripts with origin from the bi-directional promoter. In addition, a novel *CBX5* transcriptional isoform, *STET*, was discovered. This transcript includes *CBX5* exon 1 and part of intron 1 sequences through alternative splicing and polyadenylation, but lacks inclusion of HP1α encoding exons. Inverse correlation between *STET* and HP1α coding mRNA expression, transcribed from the canonical *CBX5* bi-directional promoter was observed in both breast cancer cell lines and samples from breast cancer patients. Mechanistic selection of *STET* alternative splicing and polyadenylation is expected in stoichiometric amounts to decrease the generation of *CBX5* transcripts with HP1α encoding potential. This could thereby comprise a novel mechanism of HP1α encoding mRNA regulation. However, we systematically identified *STET* mRNA in minor amounts compared to HP1α encoding mRNA. Moreover, insertion of the *STET* composite terminal exon in a mini-gene background had neither in transient nor genome integrated analysis a negative influence for the inclusion of a *STET* exon downstream located exon. Thus, we have no supportive evidence for such a regulatory model. Therefore, the results more likely reflects a model wherein reduced *CBX5* transcriptional quality mediated by promoter downstream mechanisms e.g. through impaired transcriptional re-initiation and elongation, results in relative increased inclusion of the *STET* composite exon.

### Availability of supporting data

All the supporting data are included as additional files.

## Additional files

Additional file 1: Table S1. Sequences for primers used in RT-qPCR. (DOCX 17 kb) Additional file 2: Table S2. Expression data extracted from Affymetrix microarray experiments. (DOCX 17 kb) Additional file 3: Figure S1.
*CBX1* and *CBX3* and correlation of expression analyses. A) Schematized view of *CBX3* and *hnRNPA2B1* (not drawn to scale). Arrows indicate direction of transcription. The coding region is indicated by black colouring. pA indicates the localization of poly-A signals. *A2UCOE* represents localization of the characterized insulator element. B) Correlation analysis of *CBX3* and *hnRNPA2B1* expression in the NCI-60 breast cancer cell panel. The analysis presented as heat map was performed using the CellMiner database, http://discover.nci.nih.gov/cellminer/, with red symbolizing positive and blue negative correlation. C) Correlation analysis of *CBX1*, *CBX3*, *CBX5*, *hnRNPA1* and *hnRNPA2B1* expression in the NCI-60 breast cancer cell panel. The analysis presented as correlation coefficients estimated using the CellMiner database with red numbering symbolizing significant expression correlation. D) Schematized view of *CBX1* and neighboring *SNX11* (not drawn to scale). Arrows indicate direction of transcription. The coding region is indicated by black coloring. The position of a promoter overlapping CpG island is shown. (PDF 380 kb) Additional file 4: Figure S2. TSA effects on *CBX1*, *CBX3* and *CBX5* expression. A) mRNA expression analysis of the *CBX1*, *CBX3* and *CBX5* response towards TSA. Relative expression levels of *CBX* mRNA in MCF7 and MDA-MB-231 cells after 24 h treatment with TSA or control DMSO. Relative expression was calculated from RT-qPCR using *GAPDH* expression for normalization. For all panels, bars represent mean values with standard deviations. B) Immunofluorescence analysis of HP1α (upper panels) or DAPI staining in MDA-MB-231 cells either untreated or TSA treated for 24 h. C) Zooming in on a representative immunofluorescence analysis of HP1α in MDA-MB-231 cells with arrows pointing on heterochromatic spots. (PDF 914 kb) Additional file 5: Figure S3. RNA stability analysis of *HP1α-V3* and *STET*. A-B) mRNA decay analysis of *HP1α-V3*, *STET* and *C-MYC* following Actinomycin D treatment in MCF7 (A) and MDA-MB-231 cells (B). Cells were treated with Actinomycin D and harvested at indicated time points. Relative expression was calculated from RT-qPCR using *GAPDH* expression for normalization. C) Knockdown efficiency of siRNA mediated knockdown of *RRP6* and *RRP40* mRNA in MCF7 and MDA-MB-231 cells, respectively. Expression of *HP1α-V3*, *STET* and amplicon *A1* RNA after *RRP6* and *RRP40* siRNA mediated knockdown in MCF7 and MDA-MB-231 cells, respectively. Relative expression was calculated from RT-qPCR using *GAPDH* expression for normalization. For all panels, bars represent mean values with standard deviations. (PDF 574 kb) Additional file 6: Figure S4. Expression analysis of *hnRNPA1*, *HP1α-V3* and *STET* in breast cancer biopsies normalized to normal breast biopsies. A-E) Based on a standard curve of serial dilutions of cDNA with known concentrations, quantification was determined from single measurements with the second derivate max method by the LightCycler software. Relative expression was calculated using *HMBS* for normalization. To correct for diversity of baseline expression between patients, expression of each carcinoma sample was further normalized to the corresponding normal breast tissue sample of that patient. Results are presented as log-transformed values of *HMBS* and normal breast tissue normalized data. N indicates the number of samples with measurements above limit of detection. For all panels, bars represent mean values with standard deviations. ** *P* < 0.01; one-way ANOVA with Tukey’s multiple comparison. For datasets with significantly different standard deviations between means non-parametric Kruskal-Wallis test with Dunn’s multiple comparison was performed. (PDF 333 kb)

## Abbreviations

aa: Amino acid
ac: Acetylation
APA: Alternative cleavage and polyadenylation
CBX: Chromo box protein homolog
CD: Chromo domain
ChIP: Chromatin immunoprecipitation
CoTC: Co-transcriptional cleavage
CSD: Chromoshadow domain
EGFP: Enhanced green fluorescent protein
H3: Histone 3
H3K9: Histone H3 lysine position 9
H3K9ac: Histone H3 lysine position 9 acetylation
H3K9me2/3: Histone H3 lysine position 9 di- and tri-methylation
HP1: Heterochromatin protein 1
K: Lysine
KAT: Katushka fluorescent protein
me2: Di-methylation
me3: Tri-methylation
ORF: Open reading frame
pA signal: Polyadenylation signal
PEV: Position effect variegation
Pol-II: RNA polymerase II
RT-qPCR: Reverse transcriptase quantitative PCR
SD: Standard deviation
STET: Skipped terminal exon transcript
TSA: Trichostatin-A
UTR: Untranslated region
