# Supplementary material for: Regulatory dissection of the CBX5 and hnRNPA1 bi-directional promoter in human breast cancer cells reveals novel transcript variants differentially associated with HP1α down-regulation in metastatic cells
Source: BMC Cancer. 2016 Jan 20;16:32. doi: 10.1186/s12885-016-2059-x (PMC4721113; doi:10.1186/s12885-016-2059-x)
Supplement: Additional file 1: Table S1. — Sequences for primers used in RT-qPCR. (DOCX 17 kb) [file 12885_2016_2059_MOESM1_ESM.docx]

| STable 1. Primers for RT-qPCR | | |
| --- | --- | --- |
| **Gene:** | **Forward primer (5' --> 3')** | **Reverse primer (5' --> 3')** |
| *hnRNPA1* | CCTGCCGTCATGTCTAAGT | ACAACTCTTCCATCCACCTT |
| *HP1α-pan* | GGGCAACAGATTCCTGTGGTG | CCCCTCCTTTAGCTCTTTGCTG |
| *HP1α transcript variant 1* | CCCGGAAATTCCAAGGGAC | CGCCTGTCTAGCACCTTCTC |
| *HP1α transcript variant 2* | GGGAAAGAAAGAACTGGGGC | CGCCTGTCTAGCACCTTCTC |
| *HP1α transcript variant 3* | GCAGACGTTAGCGTGAGTG | CGCCTGTCTAGCACCTTCTC |
| *STET1* | TCAATCCCGGAGAATGATTT | GTCACAGTGGGTGTTCTTCC |
| *STET2* | TCAATCCCGGGAAGGAGT | GTCACAGTGGGTGTTCTTCC |
| *STET-pan* | GCAGACGTTAGCGTGAGTG | GTCACAGTGGGTGTTCTTCC |
| *A1* | GCAGACGTTAGCGTGAGTG | CCGGGATTGAGAGTGATCAC |
| *A2* | GCAGACGTTAGCGTGAGTG | GGGTAGATAAGACTGTCTGC |
| *A3* | CCAGTAGACGGACAGCCTAA | CAAGGAGGTGTATGGGACTG |
| *A4* | ATTCGTGAACTCGGACTCTG | GTCACAGTGGGTGTTCTTCC |
| *A5* | CTGTCCAGGAGCTCTACCAA | TGAGGCTACTGCAGGATACA |
| *A6* | CAGCATTCCTTGGCATATCT | ACTCCAGACTGGGTGACAAA |
| *A7* | GTTCCATCCTGTTTCATTGC | TTGGTTTCTCCTCTCCACAG |
| *A8* | TTAAACGGGGTCTTGTTCTG | CAGCTTTTCAGAAGGCTGAG |
| *c-Myc* | TGCTTAGACGCTGGATTTCT | CCAGTCGTAGTCGAGGTCAT |
| *RRP6* | CAATGAGAGCCTCACAGACC | TGCTGCCTGATGAGTATCAA |
| *RRP40* | AGTTGATGTTGGAGGGAGTG | AACCACAAACTGGCCATAGA |
| *HMBS* | CGGTACCCACGCGAATCAC | GGGTACCCACGCGAATCAC |
| *GAPDH* | ATGGGGAAGGTGAAGGTCGGAG | GATGACAAGCTTCCCGTTCTCAGC |
| **Minigene:** |  |  |
| *hnRNPA1-EGFP/Katushka* | CCTGCCGTCATGTCTAAGT | AGACAGCACAATAACCAGCAC |
| *CBX5-EGFP/Katushka* | GCAGACGTTAGCGTGAGTG | AGACAGCACAATAACCAGCAC |
