# Supplementary material for: Regulatory dissection of the CBX5 and hnRNPA1 bi-directional promoter in human breast cancer cells reveals novel transcript variants differentially associated with HP1α down-regulation in metastatic cells
Source: BMC Cancer. 2016 Jan 20;16:32. doi: 10.1186/s12885-016-2059-x (PMC4721113; doi:10.1186/s12885-016-2059-x)
Supplement: Additional file 2: Table S2. — Expression data extracted from Affymetrix microarray experiments. (DOCX 17 kb) [file 12885_2016_2059_MOESM2_ESM.docx]

| **STable 2. Selected mRNA expression data from Affymetrix U219 microarray analysis** | | | |
| --- | --- | --- | --- |
| Gene | Affymetrix probe | Expression (log 2)  MDA-MB-231 MCF7 | |
| *CBX5* | 11716275_a_at | 3.345544 | 4.841488 |
| *CBX5* | 11716276_a_at | 6.3665 | 9.129478 |
| *CBX5* | 11716277_a_at | 4.454346 | 6.717009 |
| *CBX5* | 11716278_s_at | 5.093538 | 7.482693 |
| *CBX5* | 11716279_x_at | 5.442586 | 6.964382 |
| *CBX5* | 11716280_a_at | 4.443212 | 6.313105 |
| *CBX1* | 11715546_s_at | 9.484847 | 10.14591 |
| *CBX3* | 11715499_x_at | 10.83346 | 10.51032 |
| *CBX3* | 11754967_s_at | 11.06907 | 11.02801 |
| *CBX3* | 200037_PM_s_at | 11.34248 | 11.61323 |
| *hnRNPA1* | 11753459_x_at | 9.790039 | 11.44173 |
| *hnRNPA1* | 11757268_x_at | 10.76887 | 11.36127 |
| *hnRNPA1* | 11757837_x_at | 11.80675 | 12.02499 |
| *hnRNPA1* | 11760085_s_at | 8.653099 | 10.00078 |
| *hnRNPA1* | 200016_PM_x_at | 12.66021 | 12.86992 |
| *hnRNPA1* | 11758811_x_at | 9.259556 | 9.189816 |
| *hnRNPA1* | 11719462_x_at | 11.12534 | 12.24353 |
| *hnRNPA1* | 11756086_x_at | 8.933888 | 9.689674 |
| *hnRNPA2* | 11742679_s_at | 11.92693 | 11.72051 |
| *hnRNPA2* | 11742680_at | 7.801661 | 7.883937 |
| *GDOWN1* | 11723376_a_at | 5.099013 | 4.832868 |
| *GDOWN1* | 11723377_a_at | 5.901062 | 6.412063 |
| *GDOWN1* | 11725132_a_at | 6.981335 | 6.724336 |
| *GDOWN1* | 11725133_s_at | 8.456964 | 7.919206 |
| *GDOWN1* | 11725134_a_at | 6.50409 | 6.318328 |
| *GDOWN1* | 11741300_a_at | 6.271768 | 6.186866 |
| *GDOWN1* | 11741301_s_at | 5.714641 | 5.540685 |
| *GDOWN1* | 11741302_x_at | 5.743063 | 5.536814 |
| *HMBS* | 11720884_a_at | 8.127339 | 8.34651 |
| *GAPDH* | 11754912_x_at | 13.10566 | 12.87943 |
| *GAPDH* | AFFX-HUMGAPDH/M33197_3_at | 13.25139 | 13.06241 |
| *GAPDH* | AFFX-HUMGAPDH/M33197_5_at | 13.1645 | 13.12345 |
| *GAPDH* | AFFX-HUMGAPDH/M33197_M_at | 12.83866 | 12.73801 |
