# Supplementary material for: Regulatory dissection of the CBX5 and hnRNPA1 bi-directional promoter in human breast cancer cells reveals novel transcript variants differentially associated with HP1α down-regulation in metastatic cells
Source: BMC Cancer. 2016 Jan 20;16:32. doi: 10.1186/s12885-016-2059-x (PMC4721113; doi:10.1186/s12885-016-2059-x)
Supplement: Additional file 3: Figure S1. — CBX1 and CBX3 and correlation of expression analyses. A) Schematized view of CBX3 and hnRNPA2B1 (not drawn to scale). Arrows indicate direction of transcription. The coding region is indicated by black colouring. pA indicates the localization of poly-A signals. A2UCOE represents localization of the characterized insulator element. B) Correlation analysis of CBX3 and hnRNPA2B1 expression in the NCI-60 breast cancer cell panel. The analysis presented as heat map was performed using the CellMiner database, http://discover.nci.nih.gov/cellminer/, with red symbolizing positive and blue negative correlation. C) Correlation analysis of CBX1, CBX3, CBX5, hnRNPA1 and hnRNPA2B1 expression in the NCI-60 breast cancer cell panel. The analysis presented as correlation coefficients estimated using the CellMiner database with red numbering symbolizing significant expression correlation. D) Schematized view of CBX1 and neighboring SNX11 (not drawn to scale). Arrows indicate direction of transcription. The coding region is indicated by black coloring. The position of a promoter overlapping CpG island is shown. (PDF 380 kb) [file 12885_2016_2059_MOESM3_ESM.pdf]

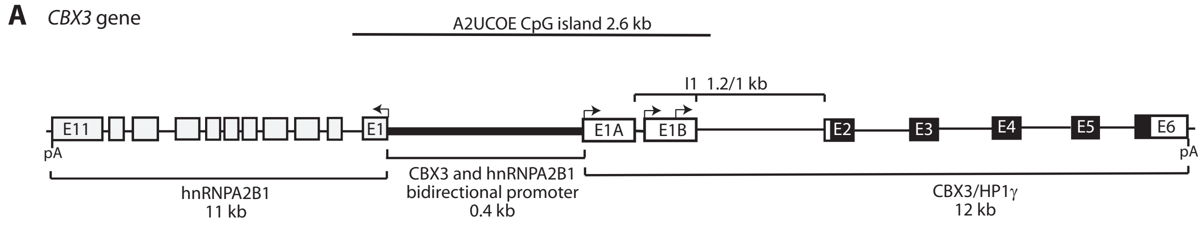

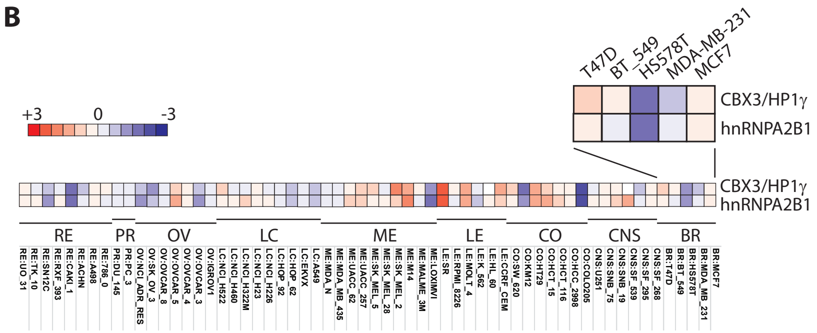

C

correlation cofficient NCI-60	CBX5/HP1α	hnRNPA1	CBX3/HP1γ	hnRNPA2B1	CBX1/HP1β
CBX5/HP1α	1	0.129	-0.005	0.012	0.231
hnRNPA1	0.129	1	0.576	0.545	-0.001
CBX3/HP1γ	-0.005	0.576	1	0.896	-0.003
hnRNPA2B1	0.012	0.545	0.896	1	-0.086
CBX1/HP1β	0.231	-0.001	-0.003	-0.086	1

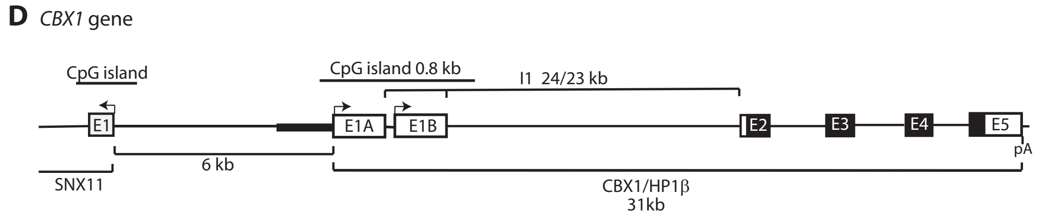

SFig. 1
