# Supplementary figures and images for: Regulatory dissection of the CBX5 and hnRNPA1 bi-directional promoter in human breast cancer cells reveals novel transcript variants differentially associated with HP1α down-regulation in metastatic cells
Source: BMC Cancer. 2016 Jan 20;16:32. doi: 10.1186/s12885-016-2059-x (PMC4721113; doi:10.1186/s12885-016-2059-x)

**A**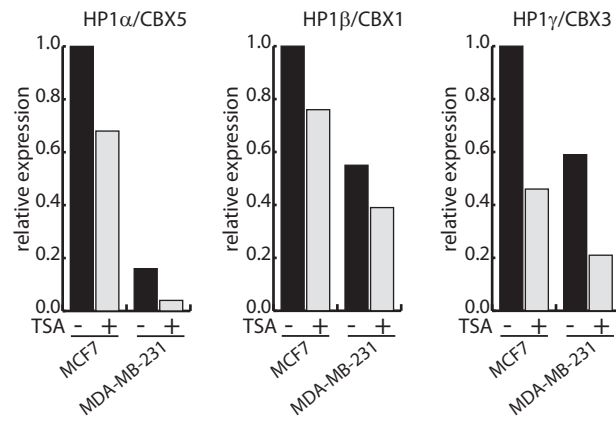**B**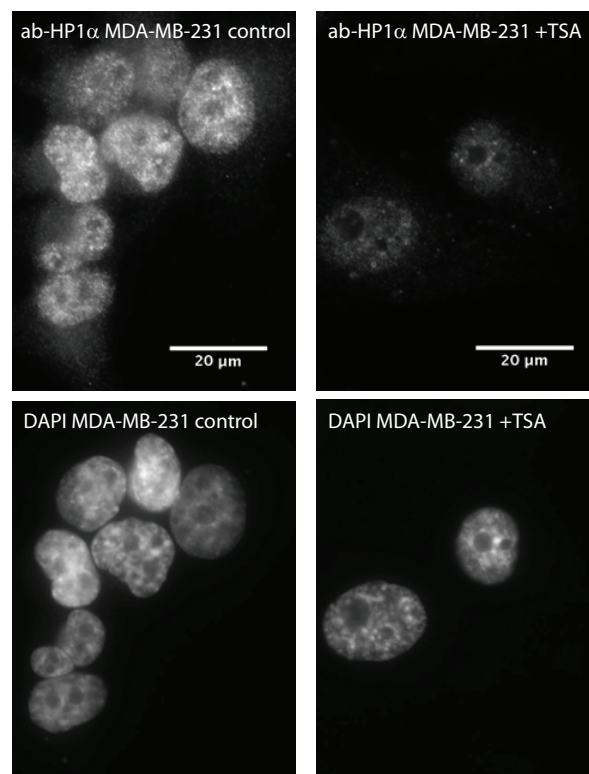**C**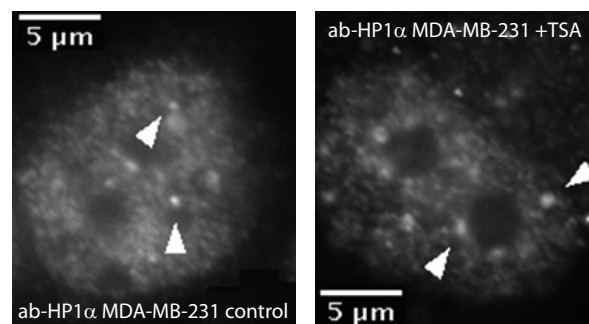

Supplement: Additional file 4: Figure S2. — TSA effects on CBX1, CBX3 and CBX5 expression. A) mRNA expression analysis of the CBX1, CBX3 and CBX5 response towards TSA. Relative expression levels of CBX mRNA in MCF7 and MDA-MB-231 cells after 24 h treatment with TSA or control DMSO. Relative expression was calculated from RT-qPCR using GAPDH expression for normalization. For all panels, bars represent mean values with standard deviations. B) Immunofluorescence analysis of HP1α (upper panels) or DAPI staining in MDA-MB-231 cells either untreated or TSA treated for 24 h. C) Zooming in on a representative immunofluorescence analysis of HP1α in MDA-MB-231 cells with arrows pointing on heterochromatic spots. (PDF 914 kb) [file 12885_2016_2059_MOESM4_ESM.pdf]

**A**

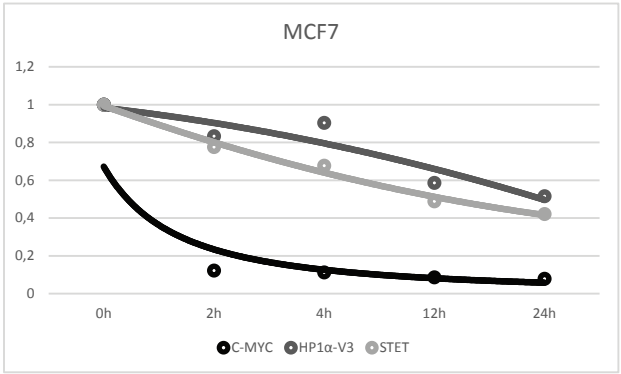

**B**

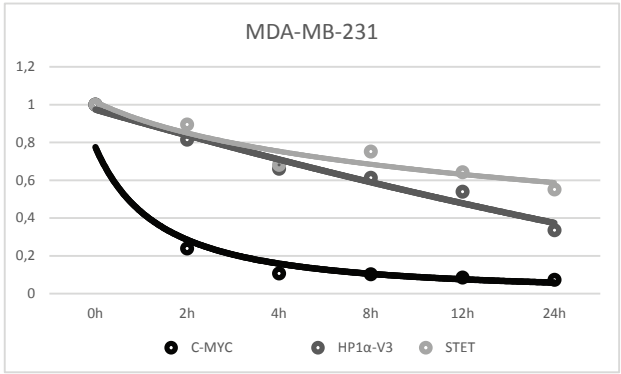

**C**

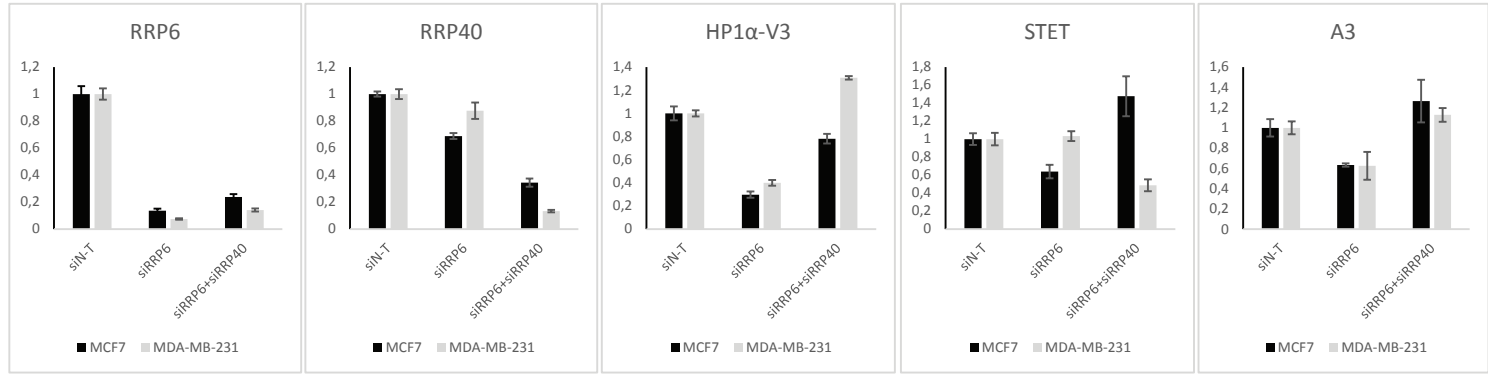

SFig. 3

Supplement: Additional file 5: Figure S3. — RNA stability analysis of HP1α-V3 and STET. A-B) mRNA decay analysis of HP1α-V3, STET and C-MYC following Actinomycin D treatment in MCF7 (A) and MDA-MB-231 cells (B). Cells were treated with Actinomycin D and harvested at indicated time points. Relative expression was calculated from RT-qPCR using GAPDH expression for normalization. C) Knockdown efficiency of siRNA mediated knockdown of RRP6 and RRP40 mRNA in MCF7 and MDA-MB-231 cells, respectively. Expression of HP1α-V3, STET and amplicon A1 RNA after RRP6 and RRP40 siRNA mediated knockdown in MCF7 and MDA-MB-231 cells, respectively. Relative expression was calculated from RT-qPCR using GAPDH expression for normalization. For all panels, bars represent mean values with standard deviations. (PDF 574 kb) [file 12885_2016_2059_MOESM5_ESM.pdf]

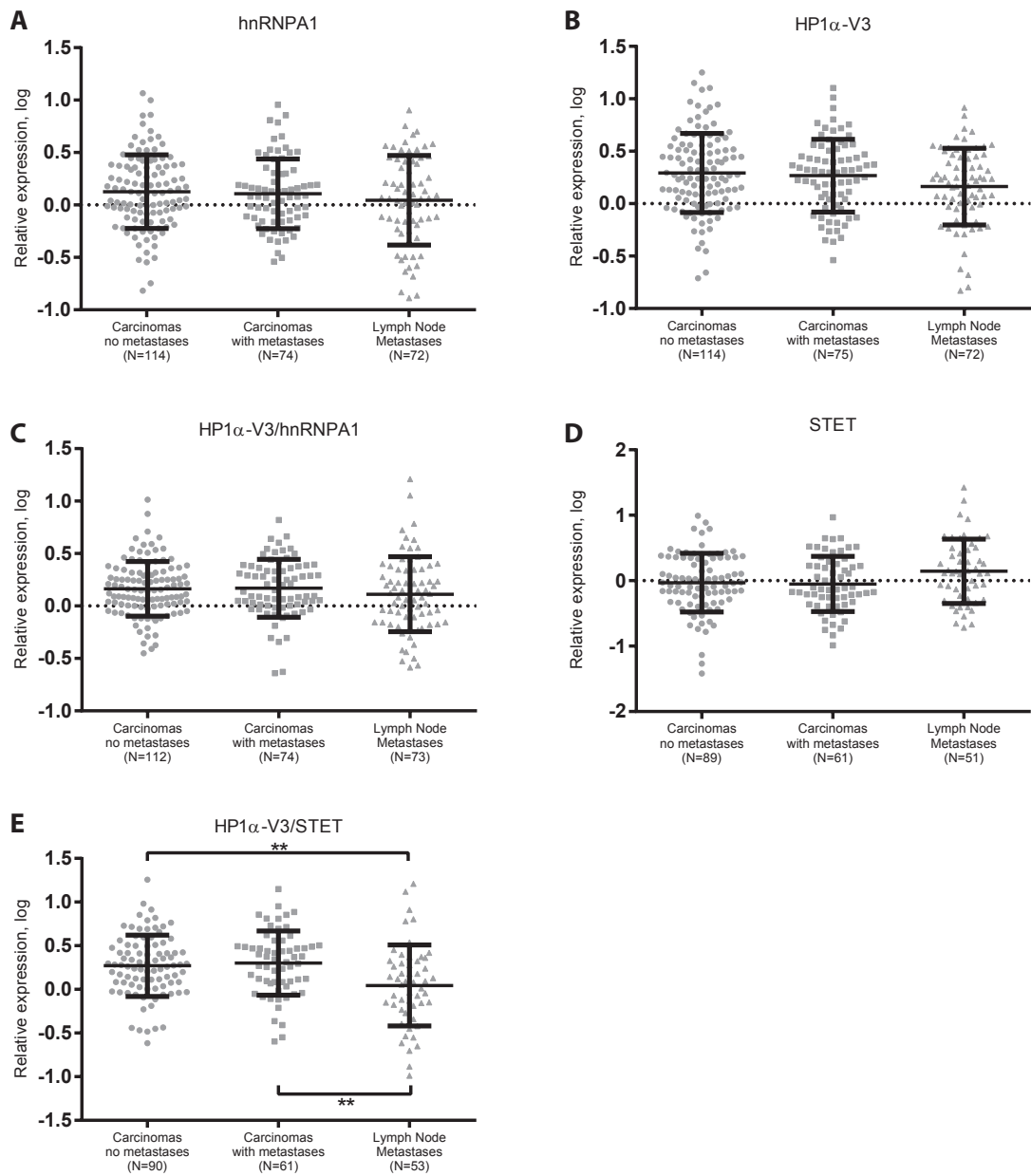

SFig. 4

Supplement: Additional file 6: Figure S4. — Expression analysis of hnRNPA1, HP1α-V3 and STET in breast cancer biopsies normalized to normal breast biopsies. A-E) Based on a standard curve of serial dilutions of cDNA with known concentrations, quantification was determined from single measurements with the second derivate max method by the LightCycler software. Relative expression was calculated using HMBS for normalization. To correct for diversity of baseline expression between patients, expression of each carcinoma sample was further normalized to the corresponding normal breast tissue sample of that patient. Results are presented as log-transformed values of HMBS and normal breast tissue normalized data. N indicates the number of samples with measurements above limit of detection. For all panels, bars represent mean values with standard deviations. ** P < 0.01; one-way ANOVA with Tukey’s multiple comparison. For datasets with significantly different standard deviations between means non-parametric Kruskal-Wallis test with Dunn’s multiple comparison was performed. (PDF 333 kb) [file 12885_2016_2059_MOESM6_ESM.pdf]
